# Major QTLs and Potential Candidate Genes for Heat Stress Tolerance Identified in Chickpea (*Cicer arietinum* L.)

**DOI:** 10.3389/fpls.2021.655103

**Published:** 2021-07-26

**Authors:** Uday Chand Jha, Harsh Nayyar, Ramesh Palakurthi, Rintu Jha, Vinod Valluri, Prasad Bajaj, Annapurna Chitikineni, Narendra P. Singh, Rajeev K. Varshney, Mahendar Thudi

**Affiliations:** ^1^Indian Council of Agricultural Research (ICAR)-Indian Institute of Pulses Research (IIPR), Kanpur, India; ^2^Department of Botany, Panjab University, Chandigarh, India; ^3^Center of Excellence in Genomics and Systems Biology (CEGSB), International Crops Research Institute for the Semi-Arid Tropics (ICRISAT), Hyderabad, India; ^4^Institute of Crop Science, Chinese Academy of Agricultural Science (CAAS), Beijing, China; ^5^State Agricultural Biotechnology Centre, Centre for Crop and Food Innovation, Food Futures Institute, Murdoch University, Murdoch, WA, Australia; ^6^University of Southern Queensland, Toowoomba, QLD, Australia

**Keywords:** chickpea, heat stress, genotyping-by-sequencing, normalized difference vegetation index, days to pod initiation

## Abstract

In the context of climate change, heat stress during the reproductive stages of chickpea (*Cicer arietinum* L.) leads to significant yield losses. In order to identify the genomic regions responsible for heat stress tolerance, a recombinant inbred line population derived from DCP 92-3 (heat sensitive) and ICCV 92944 (heat tolerant) was genotyped using the genotyping-by-sequencing approach and evaluated for two consecutive years (2017 and 2018) under normal and late sown or heat stress environments. A high-density genetic map comprising 788 single-nucleotide polymorphism markers spanning 1,125 cM was constructed. Using composite interval mapping, a total of 77 QTLs (37 major and 40 minor) were identified for 12 of 13 traits. A genomic region on CaLG07 harbors quantitative trait loci (QTLs) explaining >30% phenotypic variation for days to pod initiation, 100 seed weight, and for nitrogen balance index explaining >10% PVE. In addition, we also reported for the first time major QTLs for proxy traits (physiological traits such as chlorophyll content, nitrogen balance index, normalized difference vegetative index, and cell membrane stability). Furthermore, 32 candidate genes in the QTL regions that encode the heat shock protein genes, heat shock transcription factors, are involved in flowering time regulation as well as pollen-specific genes. The major QTLs reported in this study, after validation, may be useful in molecular breeding for developing heat-tolerant superior lines or varieties.

## Introduction

Given the global climate changes, heat stress is becoming a major challenge to crop production and food safety. As per Intergovernmental Panel on Climate Change, the current rate of global warming is 0.2°C per decade and is predicted to reach 1.5°C between 2,030 and 2,052 (https://www.bbc.com/news/newsbeat-48947573). Such an increase in temperatures leads to heat stress and costs the global economy US$2.4 trillion a year (https://news.un.org/en/story/2019/07/1041652). More than 15% of the global land area becomes exposed to high levels of heat stress with an additional 0.5°C increase to the 2°C (Sun et al., 2019). Heat stress, besides affecting producers directly, also reduces labor productivity (Kjellstrom, 2016), further compounding the effects of increasing temperature on crop yields. In recent years, shifts toward more sustainable and healthy diets, which are typically characterized by high consumption of vegetables and legumes, have been evidenced (Scheelbeek et al., 2018).

Chickpea (*Cicer arietinum* L.) is an important cool season grain legume crop cultivated in the arid and semi-arid regions across the globe. It is an excellent source of proteins, essential amino acids, vitamins, and minerals (Jukanti et al., 2012). Major chickpea producing countries are India, Australia, Pakistan, Turkey, Russia, Myanmar, Iran, Mexico, Canada, and USA. In India, Madhya Pradesh, Maharashtra, Rajasthan, Karnataka, Uttar Pradesh, and Andhra Pradesh are the major chickpea growing states. Although India is the largest producer of chickpea, in order to attain self-sufficiency by 2050, the chickpea production in the country needs to reach 16–17.5 Mt from an area of about 10.5 Mha with an average productivity of 15–17 q/ha (Dixit et al., 2019). Drought and heat, the two most important environmental factors, can cause more than 70% yield loss in chickpea (Varshney et al., 2019). Traditionally, chickpea requires prolonged winter for better growth and cultivation in the northern states of India. However, in the northern states, the pulse area especially chickpea cultivation was reduced due to the green revolution. Southern and Central India, where significant chickpea area increased, are exposed to drought and heat stresses. The rise in ambient air temperature (≥35°C) that coincides with the reproductive processes leads to various anomalies in reproductive events, especially during fertilization, pod formation, and pod filling in chickpea (Devasirvatham et al., 2013; Kaushal et al., 2013; Gaur et al., 2014).

The genetic mechanism of heat stress in different crop plants has been reviewed extensively (see Janni et al., 2020). In general, the impact of heat stress depends on the intensity, duration of exposure, and degree of the elevated temperature. In the case of legumes like chickpea, heat stress has deleterious effects on the morphology, physiology, and reproductive growth (Sita et al., 2017). The effects of heat stress on the development of various male and female tissues in different legume species have been reviewed recently (Liu et al., 2019). In the case of legume crops, heat shock proteins (*HSP*), *HSP* gene families, and various metabolites were reported to control heat stress response (see Janni et al., 2020). Heat stress adversely affects pollen viability, fertilization, and seed development, which leads to a reduced harvest index consequently, and these events greatly impact chickpea yield. In the cool season, legumes such as chickpea, lentil, faba bean, and field peas, the temperature above 30°C lead to yield losses (Jiang et al., 2015; Bishop et al., 2016; Bhandari et al., 2017). As heat stress is a complex trait governed by many genes/QTLs, breeding for heat stress tolerance in chickpea remains challenging (Krishnamurthy et al., 2011; Devasirvatham et al., 2013). Therefore, the effects of heat stress on chickpea growth, development, and yield are important to understand by observing agronomic traits to develop high-temperature-tolerant cultivars.

Genomic revolution, during the last two decades, simplified understanding of the complex responses to biotic and abiotic stress in several crop plants (Roorkiwal et al., 2020; Thudi et al., 2020). Chickpea research community has access to genome sequence (Varshney et al., 2013), genome-wide variations among diverse germplasm lines at the sequence level (Thudi et al., 2016a,b; Varshney et al., 2019) for trait dissection, and the development of climate-resilient chickpea varieties (Mannur et al., 2019; Bharadwaj et al., 2020). The genotyping-by-sequencing (GBS) approach has been extensively used for single-nucleotide polymorphism (SNP) discovery and mapping traits in several crops for genetic research and breeding applications (Chung et al., 2017), including chickpea (Jaganathan et al., 2015; Thudi et al., 2020). Besides proteomic and metabolomic approaches to understanding the molecular mechanism of heat tolerance (Parankusam et al., 2017; Salvi et al., 2018), efforts were made to map QTLs and markers associated with heat tolerance in chickpea (Thudi et al., 2014; Paul et al., 2018a; Varshney et al., 2019; Roorkiwal et al., 2020).

In this study, we reported the construction of a high-density genetic map using SNPs derived from the GBS approach and major QTLs for phenological, physiological, yield, and yield-related traits based on phenotyping of recombinant inbred line (RIL) population (DCP 92-3 × ICCV 92944) under two environments (normal and late sown) for 2 years (2017–2018 and 2018–2019). In addition, we also reported the potential candidate genes implicated for heat tolerance in the QTL regions.

## Materials and Methods

### Plant Material

A biparental mapping population, comprising 184 F_7_ RIL lines, derived from the cross DCP 92-3 × ICCV 92944 segregates for heat tolerance was used for identifying genomic regions and candidate genes for heat tolerance. DCP 92-3 is a logging and *Fusarium* wilt-resistant variety released by the Indian Council of Agricultural Research (ICAR)-Indian Institute of Pulses Research (IIPR), Kanpur, Uttar Pradesh, India for cultivation in Punjab, Haryana, Delhi, Northern Rajasthan, and Western Uttar Pradesh. Pollen viability at a critical temperature of 35°C differentiates the heat-sensitive and heat-tolerant genotypes. Based on physiological, biochemical, yield, and yield-related trait studies conducted earlier (Gaur et al., 2012; Kumar et al., 2012; Kaushal et al., 2013; Bhandari et al., 2020), the chickpea genotype ICCV 92944 was reported as a heat-tolerant genotype and was released as BARI Chola-10 in Bangladesh, as Yezin 6 in Myanmar, and as JG 14 in India and is performing well under late sown conditions.

### Phenotyping of Recombinant Inbred Line Population

In the case of chickpea, the optimal temperature for its growth ranges between 10 and 30°C. Chickpeas are sensitive to heat stress particularly at the reproductive phase (flowering and seed development). A few days of exposure to high temperatures (35°C or above) during the reproductive phase can cause heavy yield losses through flower and pod abortion. Late sowing, a simple and effective field screening technique for reproductive-stage heat tolerance in chickpea developed at the International Crops Research Institute for the Semi-Arid Tropics (ICRISAT), Patancheru, India, was adopted for phenotyping the RILs for heat stress tolerance. The F_7_ RILs (184 individuals) and parents DCP 92-3 × ICCV 92944 were evaluated for two consecutive years 2017–2018 and 2018–2019 under normal sown environment (NS; second week of November) and late sown or heat stress environment (HTS; third week of December) at ICAR-IIPR, Kanpur, Uttar Pradesh, India (26° 26′ 59.7228″ N and 80° 19′ 54.7356″ E). The experiments were conducted under field conditions in a plot admeasuring 3 × 0.6 m, and the distance between plants is 10 cm. The RIL population was evaluated in augmented block design along with the parents DCP 92-3 and ICCV 92944 and two elite chickpea genotypes JG 11 and ICC 4958. All the individuals of the population were apportioned into a total of 10 blocks along with the four checks replicated in each block. The maximum, minimum, and mean temperatures were recorded weekly during the entire cropping season for both years (Supplementary Figure 1). The mapping population was phenotyped for physiological traits like normalized difference vegetation index (NDVI; using GreenSeeker, Optical Sensor Unit, 2002 114 NTech Industries, Ukiah, USA), nitrogen balance index (NBI, using DUALEX^®^ optical leafclip meter), NBI^®^ combines chlorophyll and flavonols (related to nitrogen/carbon allocation) measured by using DUALEX^®^ optical leafclip meter, chlorophyll content (CHL, using DUALEX^®^ optical leafclip meter ng/mm^2^) and cell membrane stability (CMS, %), yield, and yield-related traits [(total filled pods per plant (FP), biological yield per plant (BYPP, g), seed yield per plant (SYPP, g), harvest index (HI, %), and 100 seed weight (100SDW, g)]. To avoid the biasness, the mean of 10 individual plants was sampled for seed yield/plant taken from each planted genotype instead of seed yield/m^2^ per plot. Furthermore, the mean of 10 plants randomly chosen from each line was used for recording the abovementioned traits for all the individuals of mapping population under NS and HTS for both years. Two irrigation and same agronomic package of practice were followed for both NS and HTS sown genotypes for both years. NDVI was measured as per the following formula NDVI = NIR–RED\NIR + RED (Myneni et al., 1995), and CMS was measured as per the formula used by Blum and Ebercon (1981). CMS = 100–membrane injury index (MII), where MII is calculated as a ratio of C1 and C2, with C1 and C2 denoting the electrolytes measured at 40 and 80°C, respectively.

### Statistical Analyses

#### Analysis of Variance, Best Linear Unbiased Prediction (BLUP), and Heritability

The ANOVA for the RIL population was performed using GenStat (17th Edition), for individual environments using the mixed model analysis. For each trait and environment, the analysis was performed considering entry and block (nested within replication) as random effects and replication as fixed effects. To pool the data across environments and to make the error variances homogeneous, the individual variances were estimated and modeled for the error distribution using the residual maximum likelihood (ReML) procedure. The *Z-*value and *F-*value were calculated for random effects and fixed effects, respectively. Broad-sense heritability was calculated as *H*^2^ = *Vg*/(*Vg* + *Ve*/*nr*), as suggested by Falconer et al. (1996), and pooled broad-sense heritability was estimated as *H*^2^ = *Vg*/{(*Vg*) + (*Vge*/*ne* +*Ve*/(*ne* × *nr*))}, as suggested by Hill et al. (2012), where *H*^2^ is the broad-sense heritability, *Vg* is the genotypic variance, *Vge* is the G × E interaction variance, *Ve* is the residual variance, *ne* is the number of environments, and *nr* is the number of replications.

#### DNA Extraction, Genotyping, and Single-Nucleotide Polymorphism Calling

DNA from 184 RILs, along with the parents, was isolated from 2-week-old seedlings following the high-throughput mini-DNA extraction method (Cuc et al., 2008). The quality of DNA was checked by using 0.8% agarose gel electrophoresis, and the quantity was assessed by Qubit^®^ 2.0 Fluorometer (Thermo Fisher Scientific Inc., USA). The GBS approach was used for SNP calling between the parents and genotyping the RILs as described by Elshire et al. (2011). GBS libraries from the parental lines and RILs were prepared using *ApeKI* endonuclease (recognition site: G/CWCG), followed by ligation with uniquely barcoded adapters using T4 DNA ligase enzyme. Such digested ligated products from each sample were mixed in equal proportion to construct the GBS libraries, which were then amplified, purified to remove excess adapters, and used for sequencing on HiSeq 2500 platform (Illumina Inc., San Diego, CA, USA). Sequence reads from raw FASTQ files were used for SNP identification and genotyping using the reference-based GBSv2 analysis pipeline implemented in TASSEL v5.0 (Bradbury et al., 2007). In brief, all reads that begin with one of the matched barcodes immediately followed by the expected four base remnants of the enzyme cut site are sorted, de-multiplexed, and trimmed to first 64 bases starting from the enzyme cut site. Reads containing N within the first 64 bases after the barcode are rejected. The remaining good quality reads (called as tags) were aligned against the draft genome sequence of chickpea using Bowtie2 software. The alignment file was then processed by using the GBSv2 analysis pipeline for SNP calling and genotyping.

### Linkage Map Construction and Identification of QTLs

In order to construct the genetic map, all markers were grouped into eight linkage groups with the logarithm of odds (LOD) threshold of 5.0. Marker order within a linkage group was assigned using the regression mapping algorithm with a maximum recombination frequency of 0.4 at a LOD of three and a jump threshold of five. The ripple command was fine-tuned by adding each marker locus to confirm the final marker order. The Kosambi mapping function was used to calculate the map distance in centimorgan (cM). The segregation distortion and chi-square (χ^2^) values were detected using JoinMap V4.0, and markers with heterozygosity and significant segregation distortion were excluded (*p* < 0.001) from the analysis. The linkage map was constructed using ICIMapping 3.2 software (Meng et al., 2015). The QTL analysis was conducted for NS 2017, NS 2018, NS pooled data, HTS 2017, HTS 2018, and HTS pooled data together with the genotyping data and genetic map information using software windows QTL Cartographer version 2.5 (Wang et al., 2012). The composite interval mapping (CIM) analysis was conducted by scanning the intervals of 1.0 cM between markers and putative QTLs with a window size of 10.0 cM and by using the parameters of model six and 1,000 times of permutation with the 0.05 significance level along with the function of “Locate QTLs” option to locate QTLs.

### Identification of Candidate Genes Within QTL Confidence Intervals

Based on the physical position of the SNPs/markers flanking the QTL regions, the candidate genes present within the determined QTL intervals were retrieved from the draft genome sequence (CaGAv1.0) of chickpea (Varshney et al., 2013). The identified genes in QTL intervals were searched against NCBI-nr protein database using BLAST program. The gene ontology (GO) terms associated with the genes were searched for GO terms, using BLAST2GO software (Conesa et al., 2005).

## Results

### Phenotypic Performance and Genetic Variability of the Parents and Mapping Population

**Figure 1:**
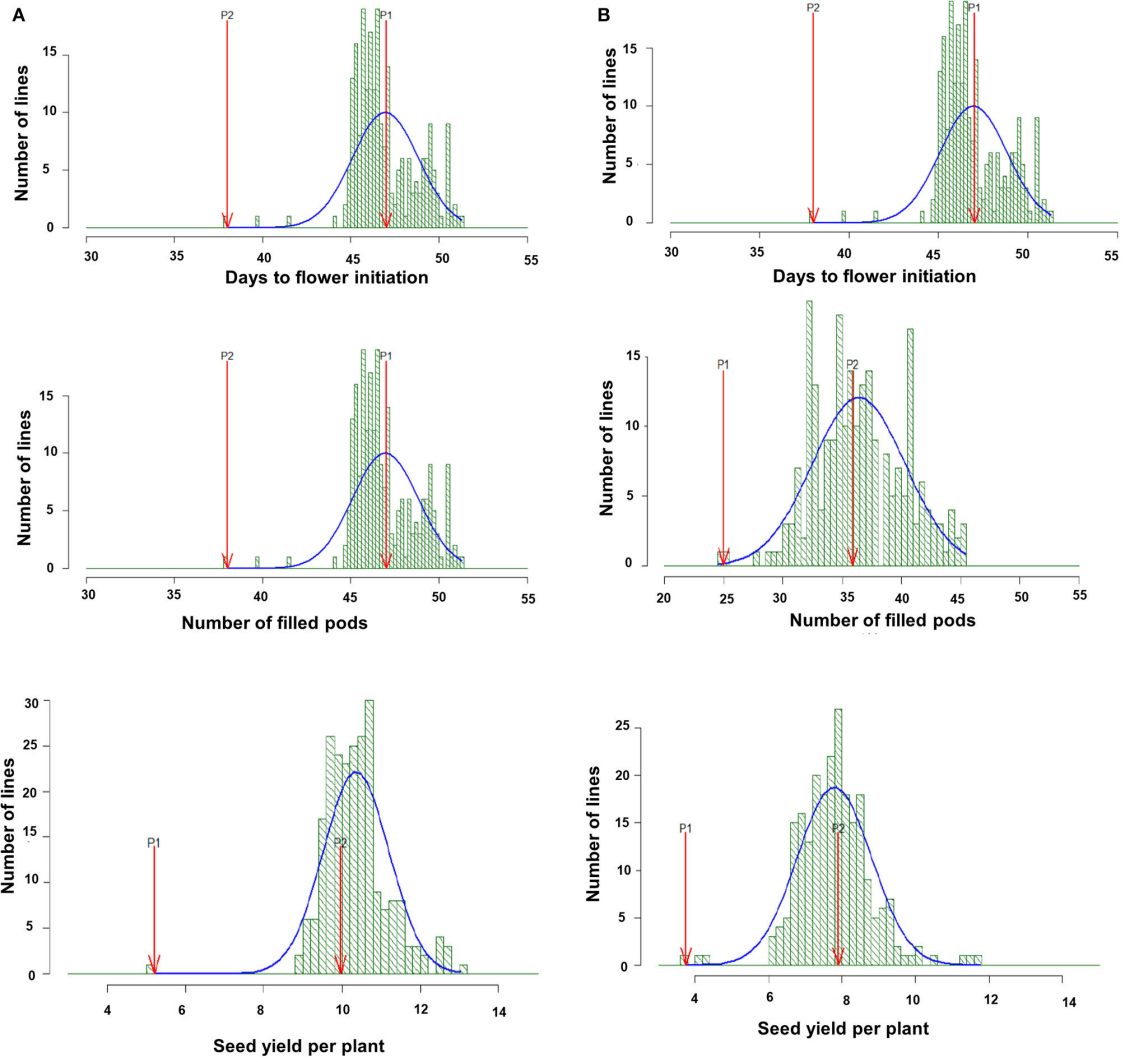
Frequency distribution of days to flower initiation (DFI, d), total filled pods per plant (FP), and seed yield per plant (SYPP, g) in RIL population derived from DCP 92-3 × ICC 92944 and evaluated, under heat stress environment 2017–18 **(A)** and under heat stress environment 2018–19 **(B)**.

A considerable amount of genetic variation for various phenological, yield, and yield-related traits was observed in both the parents and the derived RILs under NS and HTS environments for both years. The descriptive statistics are shown in Table 1. Transgressive segregates in both directions were observed for days to flower initiation (DFI), FP, and SYPP traits in the RIL population (Figure 1). The Combined ANOVA indicated the presence of significant genetic variability in the evaluated RILs under both NS and HTS. High to moderate heritability (98.2–61.3%) under NS for both years and 73.3–98.4% heritability under HTS for both years were recorded. Only low heritability of 38.2 and 47.9% for HI was observed under HTS during 2017–2018 and 2018–2019. However, high heritability (77.6–84.7%) was noted under NS conditions.

**Table 1:** Phenotypic performance of heat sensitive (DCP 92-3), tolerant (ICCV 92944), RILs, and heritability of traits evaluated under normal and heat stress environments.

**Traits**	**Environment**	**DFI (d)**	**DM (d)**	**DPI (d)**	**DPF (d)**	**NDVI**	**NBI**	**CHL (ng/ mm^**2**^)**	**CMS (%)**	**FP**	**BYPP (g)**	**100SDW (g)**	**HI (%)**	**SYPP (g)**
DCP92-3	NS 2017	56.35	136.50	74.39	22.31	0.58	23.77	24.23	46.37	72.18	31.88	15.23	65.67	21.18
	HTS 2017	49.56	103.28	65.32	11.32	0.51	23.30	22.39	38.32	41.20	19.43	13.16	41.37	7.74
	NS 2018	53.42	133.00	74.31	17.40	0.62	24.17	23.64	55.69	54.89	26.63	12.11	57.81	15.84
	HTS 2018	40.20	98.90	51.87	15.19	0.47	17.69	23.52	34.09	25.02	11.97	12.95	34.69	3.20
ICCV92944	NS 2017	45.02	118.80	60.66	18.98	0.63	22.99	25.22	53.81	82.06	45.51	26.55	67.28	31.02
	HTS 2017	51.35	101.40	62.09	11.13	0.52	22.11	25.78	43.83	47.30	17.82	17.77	54.59	9.96
	NS 2018	40.92	116.50	60.71	17.60	0.62	23.35	24.98	58.86	59.51	32.51	21.84	65.07	21.30
	HTS 2018	39.52	91.80	53.61	16.25	0.51	17.88	23.43	33.16	28.60	12.57	16.97	41.71	4.20
Means of RILs	NS 2017	57.40	127.70	78.80	18.20	0.61	17.65	30.23	56.90	88.40	35.80	21.00	66.20	25.70
	HTS 2017	47.00	97.30	60.10	12.50	0.38	18.00	28.70	45.20	44.70	21.30	20.70	49.40	10.40
	NS 2018	56.10	125.70	74.60	17.50	0.61	22.55	28.00	54.40	86.60	38.00	20.40	64.80	24.70
	HTS 2018	43.90	100.40	57.20	14.90	0.36	17.76	23.60	41.40	36.40	18.30	19.60	44.30	7.80
Range of RILs	NS 2017	53.9–59.91	124.5–130.4	74.5–82.42	12.12–24.76	0.38–0.75	11.2–25.6	17.1–45.3	44.9–66.9	79.7–97.69	29.8–41.3	14.6–31.77	57.8–71.66	20.5–31.18
	HTS 2017	39.7–51.3	94.8–100.22	51.7–64.4	8.5–19.68	0.3–0.54	14.8–24.1	15–44	42–42–50.4	38–51.12	15.8–28.22	13.7–31.85	45.7–55.69	8.84–13.04
	NS 2018	52.5–61	122.3–129.9	70.7–79.2	9–24.7	0.41–0.59	18.2–28.7	17.5–37.4	36.0–66.6	69–104.18	32.4–49.35	12.7–31.56	52.14–70.53	19.65–33
	HTS 2018	33.7–50.75	88.5–107.6	47.5–63.96	11–21.4	0.25–0.52	17.3–18	23.3–24	30.5–52.7	24.5–45.47	13.8–24.83	13–29.21	39.8–49.74	4.17–11.757
Heritability%	NS 2017	86.80	86.60	94.50	93.60	93.50	76.40	94.90	82.10	61.30	70.30	97.40	77.60	74.20
	HTS 2017	85.80	73.60	86.50	96.60	90.60	91.20	98.50	78.50	80.00	83.20	98.40	47.90	67.37
	NS 2018	87.80	83.90	92.00	98.00	77.20	80.80	88.90	92.50	80.50	77.10	98.20	84.70	73.24
	HTS 2018	92.30	95.30	91.60	90.80	90.90	86.60	80.40	68.80	84.40	83.90	96.20	38.20	77.90

*DFI, days to flower initiation; DM, days to maturity; DPI, days to pod initiation; DPF, days to pod filling; NDVI, normalized difference vegetation index; NBI, nitrogen balance index; CHL, chlorophyll content; CMS, cell membrane stability; FP, filled pods; BYPP, biological plant yield, 100SDW, 100 seed weight; HI, harvest index, SYPP, seed yield per plant*.

### Relationships Among Different Traits

To investigate the relationship among different traits, we calculated the pairwise correlations among different traits within each environment (NS and HTS). During 2017–2018, under HTS environment, a positive and high significant correlation was observed between DFI with that of days to pod initiation (DPI) (*p* < 0.01) and days to maturity (DM) (*p* < 0.01) (Supplementary Table 1). Similarly, during 2018–2019, under HTS environment, a positive and high significant correlation was also observed between DFI with that of DPI (*p* < 0.01) and DM (*p* < 0.05) (Supplementary Table 2). Furthermore, during 2017–2018, under HTS environment, NBI and CHL were found to possess a positive and high significant correlation (*p* < 0.01). However, no significant correlation was observed during 2018–2019 under HTS environment. A number of filled pods (NFP) and SYPP had a significant and positive correlation under heat stress environments during both years. Furthermore, NPF has a significant positive correlation with BYPP in 2017–2018 and with HI in 2018–2019 (Supplementary Tables 1, 2). Nevertheless, 100SDW possesses a positive and high significant correlation with HI and SYPP during both years under HTS environment (Supplementary Tables 1, 2). Similar positive and high significant correlations were also observed under NS environments in both years as well as pooled data of NS environments for the abovementioned traits (Supplementary Tables 3–6).

### Single-Nucleotide Polymorphisms-Based Genetic Map

**Figure 2:**
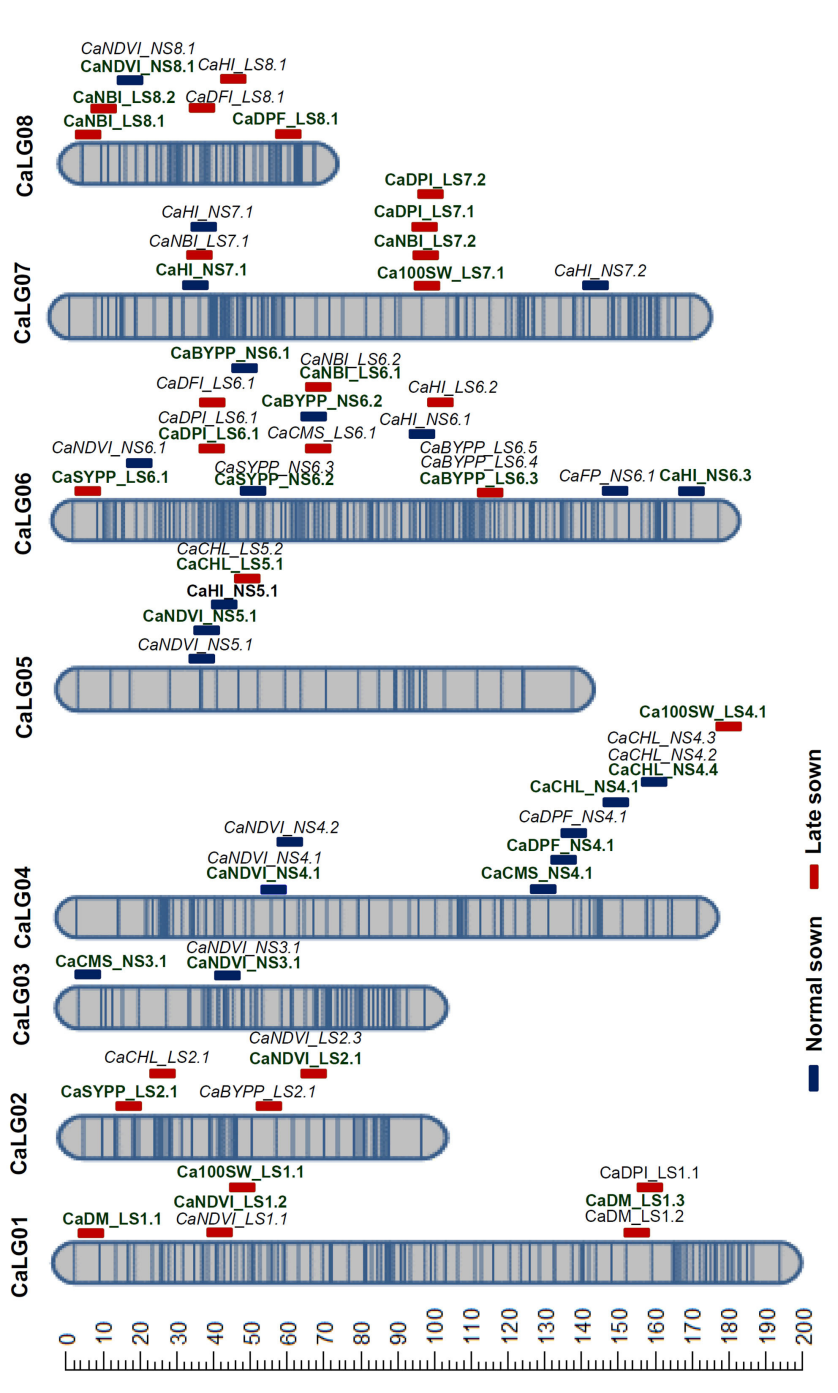
Genetic map and QTLs for heat tolerance-related traits. A genetic map comprising 788 SNPs distributed in eight linkage groups (CaLG01–CaLG08) spanning 1,125 cM was constructed using RIL population derived from DCP 92-3 × ICCV 92944. The blue and red-colored bars indicate the QTLs identified under normal and late sown conditions, respectively. The major QTLs are indicated in bold font and the minor QTLs in italics.

A total of 49.89 Gb (49 million reads) clean GBS reads were generated using HiSeq2500 on the RIL population derived from DCP 92-3 × ICCV 92944. The number of reads generated per individual ranged from 0.86 to 5.3 million. A total of 3,425,458 genome-wide SNPs were identified on aligning the data to CDC Frontier reference genome (Varshney et al., 2013) using TASSEL-GBS pipeline. After excluding ambiguous SNP calls, SNPs that are monomorphic among the parental genotypes, and SNPs with segregation distortion, a total of 7,947 polymorphic SNPs were used for the linkage map analysis using ICIM. As a result, a genetic map comprising 788 SNPs distributed on eight linkage groups (CaLG01–CaLG08) spanning 1,125 cM was constructed (Figure 2; Supplementary Tables 7, 8; Supplementary Figure 2). The CaLG06 had the highest proportion of the mapped SNPs (23.4%; 185 SNPs), whereas CaLG08 had the lowest proportion of the mapped SNPs (7.6%; 60 SNPs) and the largest linkage group CaLG01 spanned 191 cM, whereas the smallest linkage group CaLG08 spanned 68 cM.

### QTLs for Heat Stress Tolerance Traits

By using CIM, a total of 77 QTLs (37 major QTLs and 40 minor QTLs) were identified for 12 of 13 traits phenotyped for two seasons (2017–2018 and 2018–2019) and two environments (NS and HTS). Of 77 QTLs, 37 QTLs were major explaining ≥10% phenotypic variation (PVE), and 40 QTLs were minor explaining <10% PVE (Table 2). A positive value of the additive variance of a given QTL indicates that the female parent (DCP 92-3) has a positive effect on the trait; while a negative value indicates that the male parent (ICCV 92944) having a positive effect on the trait.

**Table 2:** Summary of QTLs identified for phenological, physiological, yield, and yield-related traits using RIL population derived from DCP 92-3 × ICCV 92944.

**Trait**	**Season**	**Environment**	**Linkage group**	**QTL name**	**Position (cM)**	**Left marker**	**Right marker**	**PVE%**	**Additive effect**
**PHENOLOGICAL TRAITS**									
Days to flowering initiation (DFI)	2018–19	Late sown	CaLG06	CaDFI_LS6.1	37.11	SCA6_43908965	SCA6_39028647	8.01	−2.94
	2018–19	Late sown	CaLG08	CaDFI_LS8.1	42.71	SCA8_7197652	SCA8_14126483	7.80	−1.33
	Pooled	Late sown	CaLG06	CaDFI_LS6.1	37.11	SCA6_43908965	SCA6_39028647	8.96	−2.85
	Pooled	Late sown	CaLG08	CaDFI_LS8.1	42.71	SCA8_7197652	SCA8_14126483	7.48	−1.15
Days to maturity (DM)	2017–18	Late sown	CaLG01	CaDM_LS1.1	7.11	SCA1_888	SCA1_30956998	18.13	0.98
	2017–18	Late sown	CaLG01	CaDM_LS1.2	152.61	SCA1_19586410	SCA1_19572921	8.96	−0.57
	2017–18	Late sown	CaLG01	CaDM_LS1.3	154.81	SCA1_11502160	SCA1_19572921	15.78	−0.84
**PHYSIOLOGICAL TRAITS**									
Chlorophyll content (CHL, ng/ mm^2^)	2017–18	Normal sown	CaLG04	CaCHL_NS4.3	151.51	SCA4_14861717	SCA4_48715028	33.52	4.12
	2017–18	Normal sown	CaLG04	CaCHL_NS4.3	151.51	SCA4_48714912	SCA4_48720330	8.26	3.31
	2018–19	Late sown	CaLG02	CaCHL_LS2.1	38.31	SCA2_30364073	SCA2_30370411	8.14	−0.03
	Pooled	Late sown	CaLG05	CaCHL_LS5.1	44.01	SCA5_1154130	SCA5_30627756	15.04	3.81
	Pooled	Late sown	CaLG05	CaCHL_LS5.2	44.31	SCA5_11665932	SCA5_1154130	6.78	4.43
	Pooled	Normal sown	CaLG04	CaCHL_NS4.1	142.91	SCA4_14861717	SCA4_48715028	19.71	2.92
	Pooled	Normal sown	CaLG04	CaCHL_NS4.2	150.11	SCA4_48715028	SCA4_48714912	9.92	2.74
Cell membrane stability (CMS, %)	2017–18	Normal sown	CaLG04	CaCMS_NS4.1	133.61	SCA4_48720031	SCA4_11271232	10.33	1.91
	Pooled	Late sown	CaLG06	CaCMS_LS6.1	67.21	SCA6_10020187	SCA6_10626699	7.75	2.86
	Pooled	Normal sown	CaLG03	CaCMS_NS3.1	0.01	SCA3_8852605	SCA3_9063118	11.37	−3.66
Nitrogen balance index (NBI)	2017–18	Late sown	CaLG08	CaNBI_LS8.3	3.81	SCA8_11012719	SCA8_6301805	11.44	1.35
	2017–18	Late sown	CaLG08	CaNBI_LS8.1	0.01	SCA8_6301805	SCA8_15284963	9.95	1.01
	2018–19	Late sown	CaLG08	CaNBI_LS8.2	1.01	SCA8_6301805	SCA8_15284963	13.93	−0.05
	2018–19	Late sown	CaLG07	CaNBI_LS7.2	97.01	SCA7_47907019	SCA7_9555338	11.94	−0.18
	2018–19	Late sown	CaLG07	CaNBI_LS7.1	34.61	SCA7_44149643	SCA7_28235343	9.31	0.05
	Pooled	Late sown	CaLG08	CaNBI_LS8.2	1.01	SCA8_6301805	SCA8_15284963	11.46	2.20
	Pooled	Late sown	CaLG06	CaNBI_LS6.1	69.71	SCA6_10671035	SCA6_10020177	10.26	1.51
	Pooled	Late sown	CaLG08	CaNBI_LS8.1	0.01	SCA8_6301805	SCA8_15284963	9.95	1.92
	Pooled	Late sown	CaLG06	CaNBI_LS6.2	70.71	SCA6_10671035	SCA6_10020177	8.96	1.42
	Pooled	Late sown	CaLG07	CaNBI_LS7.1	34.61	SCA7_44149643	SCA7_28235343	7.39	−2.16
Normalized difference vegetation index (NDVI)	2017–18	Late sown	CaLG02	CaNDVI_LS2.1	65.41	SCA2_31975221	SCA2_8484804	26.31	0.03
	2017–18	Late sown	CaLG02	CaNDVI_LS2.2	66.01	SCA2_16462107	SCA2_31975187	8.84	0.03
	2017–18	Normal sown	CaLG04	CaNDVI_NS4.1	68.31	SCA4_16039554	SCA4_15942274	11.45	0.05
	2017–18	Normal sown	CaLG04	CaNDVI_NS4.2	69.21	SCA4_47389419	SCA4_15935131	7.03	0.05
	2018–19	Late sown	CaLG02	CaNDVI_LS2.1	65.41	SCA2_31975221	SCA2_8484804	22.94	0.03
	2018–19	Late sown	CaLG02	CaNDVI_LS2.2	66.01	SCA2_16462107	SCA2_31975187	9.85	0.04
	2018–19	Normal sown	CaLG03	CaNDVI_NS3.1	48.41	SCA3_4871529	SCA3_18799532	10.73	0.00
	2018–19	Normal sown	CaLG08	CaNDVI_NS8.1	18.61	SCA8_11729896	SCA8_12875512	10.27	0.00
	2018–19	Normal sown	CaLG08	CaNDVI_NS8.2	18.91	SCA8_11269673	SCA8_11729896	9.21	0.00
	2018–19	Normal sown	CaLG03	CaNDVI_NS3.1	48.41	SCA3_4871529	SCA3_18799532	9.11	0.00
	2018–19	Normal sown	CaLG06	CaNDVI_NS6.1	20.01	SCA6_57720446	SCA6_57760410	6.69	0.00
	Pooled	Late sown	CaLG01	CaNDVI_LS1.2	44.21	SCA1_8682204	SCA1_33504088	34.02	−0.06
	Pooled	Late sown	CaLG01	CaNDVI_LS1.1	42.21	SCA1_33504088	SCA1_40495126	9.39	−0.06
	Pooled	Normal sown	CaLG05	CaNDVI_NS5.2	36.11	SCA5_12124749	SCA5_22672234	10.40	0.04
	Pooled	Normal sown	CaLG05	CaNDVI_NS5.1	35.11	SCA5_12124749	SCA5_22672234	9.47	0.04
	Pooled	Normal sown	CaLG04	CaNDVI_NS4.1	68.31	SCA4_16039554	SCA4_15942274	7.41	0.03
**YIELD AND YIELD RELATED TRAITS**									
Days to pod initiation (DPI, d)	2017–18	Late sown	CaLG07	CaDPI_LS7.2	98.01	SCA7_47907019	SCA7_9555338	43.49	−1.38
	Pooled	Late sown	CaLG07	CaDPI_LS7.1	97.01	SCA7_47907019	SCA7_9555338	10.52	−4.80
	Pooled	Late sown	CaLG06	CaDPI_LS6.1	37.11	SCA6_43908965	SCA6_39028647	10.33	−2.75
	2017–18	Late sown	CaLG06	CaDPI_LS6.1	37.11	SCA6_43908965	SCA6_39028647	5.88	−1.72
	2018–19	Late sown	CaLG06	CaDPI_LS6.1	37.11	SCA6_43908965	SCA6_39028647	8.45	−2.71
	Pooled	Late sown	CaLG01	CaDPI_LS1.1	153.61	SCA1_19586410	SCA1_19572921	8.14	−1.03
Days to pod filling (DPF, d)	2018–19	Late sown	CaLG08	CaDPF_LS8.1	67.41	SCA8_1742959	SCA8_3665619	11.97	−1.06
	Pooled	Normal sown	CaLG04	CaDPF_NS4.2	136.61	SCA4_48657505	SCA4_48720031	11.96	1.29
	Pooled	Normal sown	CaLG04	CaDPF_NS4.1	138.11	SCA4_48714724	SCA4_48657505	9.38	1.10
Number of filled pods (FP)	Pooled	Normal sown	CaLG06	CaFP_NS6.1	141.40	SCA6_34028484	SCA6_36622908	6.60	3.41
100 seed weight (100SW, g)	2017–18	Late sown	CaLG07	Ca100SW_LS7.1	97.01	SCA7_47907019	SCA7_9555338	31.30	4.33
	Pooled	Late sown	CaLG01	Ca100SW_LS1.1	46.21	SCA1_8682204	SCA1_33504088	37.23	2.73
	Pooled	Late sown	CaLG04	Ca100SW_LS4.1	159.71	SCA4_40568556	SCA4_14861717	36.34	2.85
	Pooled	Late sown	CaLG07	Ca100SW_LS7.1	97.01	SCA7_47907019	SCA7_9555338	33.48	4.11
Seed yield/plant (SYPP, g)	2018–19	Late sown	CaLG02	CaSYPP_LS2.1	22.51	SCA2_22704770	SCA2_35770691	18.00	−0.50
	2018–19	Late sown	CaLG06	CaSYPP_LS6.1	12.21	SCA6_35796441	SCA6_2512179	13.97	−1.60
	2018–19	Normal sown	CaLG06	CaSYPP_NS6.2	52.31	SCA6_9908036	SCA6_10234443	11.88	1.60
	2018–19	Normal sown	CaLG06	CaSYPP_NS6.3	53.01	SCA6_9993257	SCA6_9908036	8.66	1.38
Biological yield/plant (BYPP, g)	2017–18	Late sown	CaLG06	CaBYPP_LS6.5	115.31	SCA6_7929647	SCA6_7939281	10.79	1.24
	2018–19	Normal sown	CaLG06	CaBYPP_NS6.1	52.31	SCA6_9908036	SCA6_10234443	11.16	1.72
	Pooled	Normal sown	CaLG06	CaBYPP_NS6.1	52.31	SCA6_9908036	SCA6_10234443	10.70	2.16
	2017–18	Late sown	CaLG06	CaBYPP_LS6.3	114.01	SCA6_7939281	SCA6_7929339	9.37	1.14
	2018–19	Late sown	CaLG02	CaBYPP_LS2.1	55.91	SCA2_35860429	SCA2_29590953	7.23	−2.11
	2018–19	Late sown	CaLG06	CaBYPP_LS6.4	115.01	SCA6_7939281	SCA6_7929339	6.92	0.67
	Pooled	Late sown	CaLG06	CaBYPP_LS6.5	115.31	SCA6_7929647	SCA6_7939281	7.46	0.94
	2018–19	Normal sown	CaLG06	CaBYPP_NS6.2	58.71	SCA6_10672468	SCA6_10231199	9.43	1.56
Harvest index (HI, %)	2017–18	Normal sown	CaLG05	CaHI_NS5.1	42.11	SCA5_30627756	SCA5_41304451	18.69	2.30
	2017–18	Normal sown	CaLG07	CaHI_NS7.1	35.81	SCA7_36854123	SCA7_44149692	12.38	−2.53
	2018–19	Normal sown	CaLG06	CaBYPP_NS6.3	170.81	SCA6_52007475	SCA6_44667261	39.31	−2.26
	2018–19	Late sown	CaLG06	CaHI_LS6.2	100.21	SCA6_8170633	SCA6_7835024	7.31	−0.62
	Pooled	Late sown	CaLG08	CaHI_LS8.1	43.11	SCA8_14325980	SCA8_7197652	7.10	2.02
	2017–18	Normal sown	CaLG07	CaHI_NS7.2	142.71	SCA7_42355015	SCA7_30768244	9.24	3.24
	2018–19	Normal sown	CaLG06	CaHI_NS6.1	84.21	SCA6_7722925	SCA6_9536577	8.08	−1.69
	Pooled	Normal sown	CaLG07	CaHI_NS7.1	35.81	SCA7_36854123	SCA7_44149692	8.92	−2.40

*PVE (%) = percent phenotypic variation explained; a positive value means the female parent (DCP92-3) having a positive effect on the trait. A negative value means the male parent (ICCV 92944) having a positive effect on the trait*.

*PVE (%) = percent phenotypic variation explained; a positive value means the female parent (DCP92-3) having a positive effect on the trait. A negative value means the male parent (ICCV92944) having a positive effect on the trait*.

### QTLs for Phenological Traits

**Figure 3:**
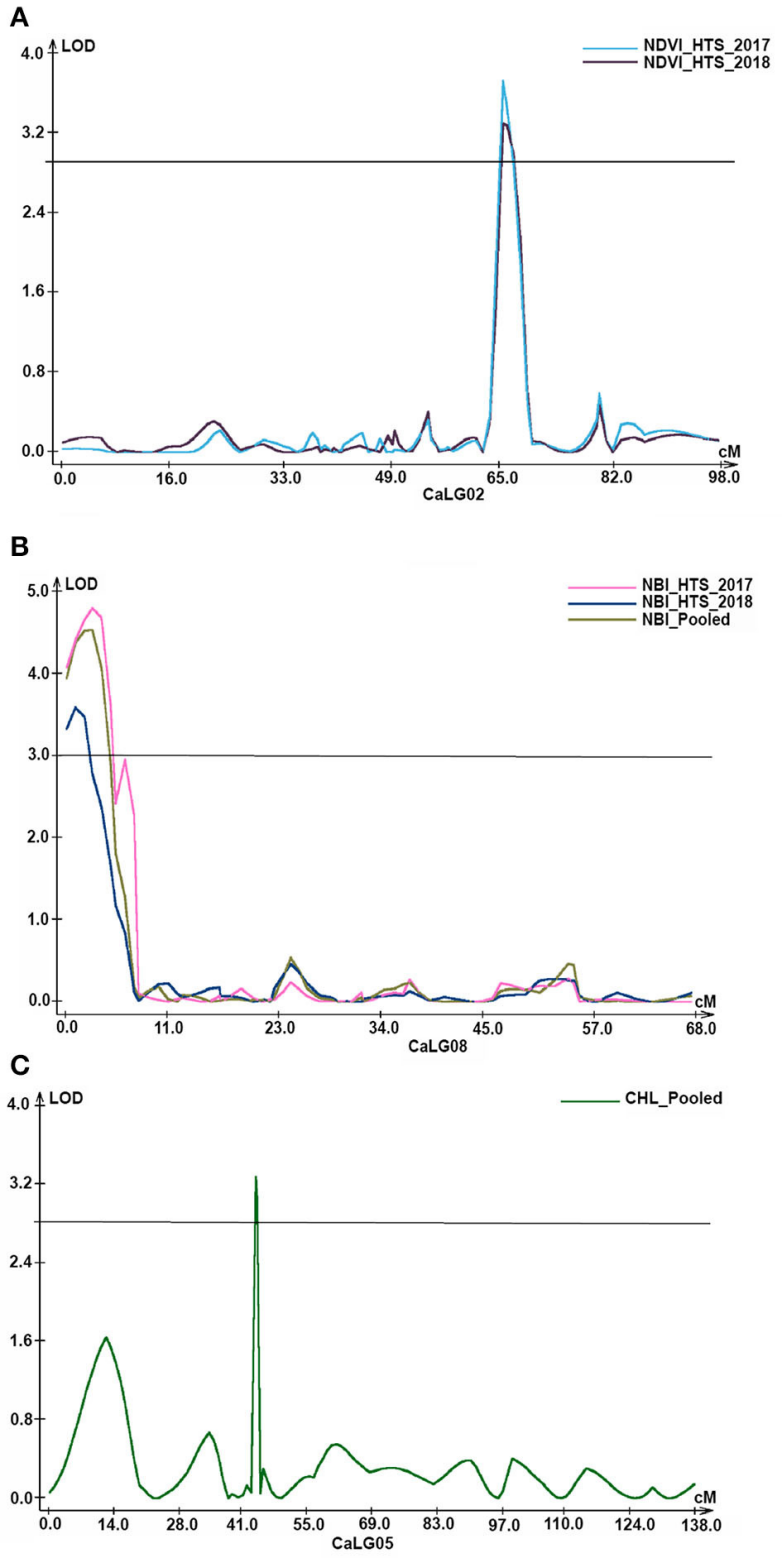
Genomic regions with major QTLs for physiological traits. **(A)** Under heat stress environments of 2017–18 and 2018–19, two QTLs explaining 8.84% and 9.85% PVE, respectively, for normalized difference vegetation index (NDVI) were identified on CaLG02; **(B)** similarly, in addition to QTLs under two heat stress environments, QTLs for nitrogen balance index (NBI) were also identified based on pooled data on CaLG08; **(C)** a major QTL explaining 15.04% PVE for chlorophyll content on CaLG05 based on pooled data of under heat stress environments of 2017–18 and 2018–19.

Under the HTS environment, in the case of DFI, two QTLs each were identified during 2018–2019 and pooled data of 2017–2018 and 2018–2019 on CaLG06 and CaLG08. The PVE ranged from 7.48 to 8.96%. While in the case of DM, all three QTLs (PVE 8.96–18.13%) identified were in 2017–2018 and under HTS environment on CaLG01. In the case of DFI, an additive effect for QTLs on CaLG06 ranged from −2.84 to −2.94 (Table 2; Figure 3).

### QTLs for Physiological Traits

A total of 36 (17 major and 19 minor) QTLs were identified for physiological traits with PVE ranging from 6.69% to 34.02%.

#### Normalized Difference Vegetation Index (NDVI)

A total of 16 QTLs (seven major with PVE 10.27–34.02% and nine minor with PVE 6.69–9.85%) were identified for NDVI, out of which six were identified based on HTS environment and 10 were identified based on NS environment. Interestingly, for this trait, QTLs were identified on all linkage groups except CaLG07. Furthermore, the majority of QTLs (25%) were present on CaLG02 that explained 8.84–26.31% PVE. However, the QTL on CaLG01 that explained the highest PVE (34.02%) among all QTLs identified for this trait was based on pooled HTS environment (Figure 3A).

#### Nitrogen Balance Index (NBI)

A total of 10 QTLs (five major with PVE 10.26–13.93% and five minor with PVE 7.39–9.95%) were identified for NBI. Among these 10 QTLs, five were on CaLG08, three on CaLG07, and two on CaLG06. Of five QTLs identified on CaLG08, four QTLs were flanked by SCA8_6301805 and SCA8_11012719 markers and one QTL was flanked by SCA8_6301805–SCA8_11012719 markers. Furthermore, among these five QTLs, two each were identified based on the pooled data from HTS environments and HTS of 2018–2019 and one based on HTS 2017–2018 (Figure 3B).

#### Chlorophyll Content (CHL)

Of the seven QTLs identified for CHL, four QTLs were on CaLG04, two were on CaLG05 and one on CaLG02. Under HTS 2018, one minor QTL (PVE 8.14%) was identified on CaLG02. Similarly, under HTS pooled data, one major QTL (PVE 15.04%) and one minor QTL (PVE 6.78%) were identified for CHL on CaLG05 flanked by SCA5_30627756–SCA5_1154130 and SCA5_1154130–SCA5_11665932 markers, respectively (Table 2; Figure 3C). Furthermore, under NS 2017, one major QTL (PVE 33.52%) and one minor QTL (PVE 8.26%) were identified for CHL on CaLG04 flanked by SCA4_48715028–SCA4_14861717 and SCA4_48720330–SCA4_48714912 markers, respectively. In addition, one major QTL (PVE 19.71%) and one minor QTL (PVE 9.92%) were identified on CaLG04 based on pooled data under NS.

#### Cell Membrane Stability (CMS)

Of three QTLs identified for CMS, one QTL each was on CaLG03, CaLG04, and CaLG06. Among these QTLs, two were identified based on pooled data from the NS environment and one based on pooled data from the HTS environment.

### QTLs for Yield and Yield-Related Traits

Eighteen major and 16 minor QTLs were identified for yield and yield-related traits with PVE ranging from 5.88 to 43.49% (Table 2).

#### Days to Pod Initiation

**Figure 4:**
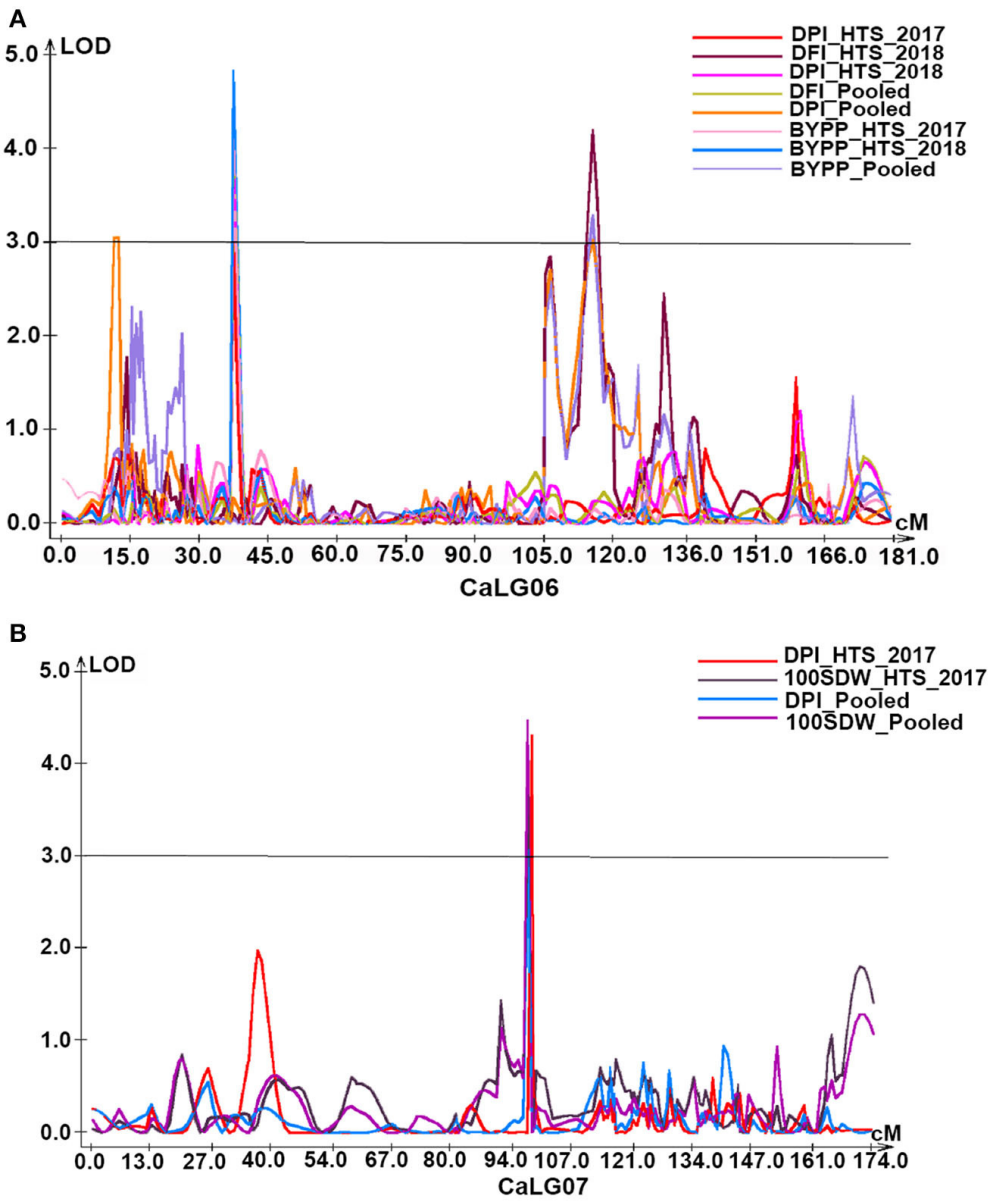
Genomic regions with major QTLs for yield and yield-related traits. **(A)** Under heat stress environments of 2017–18 and 2018–19 as well as based on pooled data of these two environments QTLs for days to pod initiation (DPI, d) and biological yield per plant (BYPP, g) were mapped on CaLG06. In addition, days to flower initiation (DPI, d) of two QTLs for normalized difference vegetation index (NDVI) were identified explaining 8.84% and 9.85% PVE, respectively, on CaLG02; **(B)** QTLs for days to pod initiation (DPI, d) and 100 seed weight (100SDW, g) under stress environment of 2017–18 as well as based on pooled data of both heat stress environments of 2017–18 and 2018–19 were co-localized on CaLG07.

Under HTS environments (2017–2018, 2018–2019, and pooled data), a total of 6 QTLs (three major with PVE 10.33–43.49% and three minor with PVE 5.88–8.45%) were identified for DPI. Out of these, one QTL was on CaLG01, three on CaLG06, and two on CaLG07. Furthermore, all QTLs identified on CaLG06 were flanked by SCA6_39028647–SCA6_43908965 markers, while QTLs on CaLG07 were flanked by SCA7_9555338–SCA7_47907019 markers. However, the QTL explaining the highest proportion of PVE was present on CaLG07 (Figure 4).

#### Days to Pod Filling and Number of Filled Pods

Of three QTLs (two major with PVE 11.96–11.97% and one minor with PVE 9.38%), two were on CaLG04 and one was on CaLG08. The major QTL on CaLG08 was identified under HTS 2018, while the remaining two were based on the pooled data of the NS environment. However, all three QTLs were flanked by different markers (Table 2). One minor QTL (PVE 6.6%) for NFP was identified on CaLG06 based on the pooled data of the NS environment.

#### Seed Yield per Plant (g)

A total of four QTLs (three major with PVE 11.88–18% and one minor with PVE 8.66%) were identified for SYPP, of which two major QTLs were under the HTS 2018 environments and the remaining two were based on pooled data of the NS environment.

#### Biological Yield per Plant (g)

A total of eight QTLs (three major with PVE 10.7–11.16% and five minor with PVE 6.92–9.43%) were identified for BYPP. Of eight QTLs, five were identified in the HTS environments of 2017–2018 and 2018–2019 and based on pooled data, and three were identified in the NS environments and based on pooled data. Among these QTLs, seven QTLs were present on CaLG06 and one on CaLG02. Furthermore, all eight QTLs were flanked by different markers (Table 2; Figure 4A).

#### Harvest Index (%)

Of eight QTLs, one minor QTL each was identified for HI under the HTS environment of 2018 and pooled data of both years, while the remaining six QTLs were on the NS environments of 2017–18 and 2018–19 and pooled data of both years. Among six QTLs under the NS environments, three QTLs were major with PVE 12.38–39.31% and three were minor with PVE 8.08–9.24%. Furthermore, among eight QTLs, three QTLs were located on CaLG06, three on CaLG07, and one each on CaLG05 and CaLG08.

#### 100 Seed Weight (g)

A total of three major QTLs were identified for 100SDW under HTS 2017 (one QTL) and based on pooled data (three QTLs) under HTS environments for both years. Among four QTLs, two were located on CaLG07 (Figure 4B), while one each was located on CaLG01 and CaLG04. Furthermore, the PVE for these four QTLs ranged from 31.3 to 37.23%.

### Candidate Genes in QTL Regions

Mining of the candidate genes for heat tolerance revealed 1,498 genes in 24.82 Mb (8.68–33.50 Mb) region on CaLG01, 1,162 genes in 23.49 Mb (8.48–31.98 Mb) region on CaLG02, 1,408 genes in 25.71 Mb (14.86–40.57 Mb) region on CaLG04, 140 genes in 4.88 Mb (39.03–43.91 Mb) region on CaLG06, and 2,074 genes in 38.35 Mb (9.56–47.91 Mb) region on CaLG07 (Supplementary Table 9). Based on functional categorization, many genes were found to be associated with biological processes in these genomic regions. Using GO classification, we further identified a total of 32 candidate genes (7 on CaLG01, 3 on CaLG02, 14 on CaLG04, and 8 on CaLG07) known to function, directly or indirectly, as heat–stress response genes in chickpea (Table 3). Among seven genes on CaLG01, six genes were encoding heat shock proteins, while one gene was encoding pollen-specific leucine-rich repeat extensin-like protein 1. While in the case of CaLG02, of three selected candidate genes, Ca_16007 encoded pollen-specific leucine-rich repeat extensin-like protein 1, Ca_24649 encoded a truncated transcription factor CAULIFLOWER A-like, and Ca_22033 encoded heat shock protein-binding protein. Among 14 selected candidate genes on CaLG04, six genes were pollen-specific, four were related to heat shock protein, three were DnaJ heat shock amino-terminal domain protein, and one was related to protein PHOTOPERIOD-INDEPENDENT EARLY FLOWERING 1 isoform X1 (Table 3). The eight genes on CaLG04 encode heat shock protein/heat shock factor protein HSF24-like (Ca_18924, Ca_16239, and Ca_09277), pollen-specific leucine-rich repeat extensin-like protein 1/pollen receptor-like kinase 3 (Ca_16434 and Ca_16155), protein EARLY FLOWERING 3/flowering time control protein FY (Ca_10118 and Ca_17996), and calmodulin-binding heat-shock protein (Ca_13761) (Table 3).

**Table 3:** Key heat stress responsive genes in the QTL regions.

**Gene**				**Pseudomolecule**		**Sequence description**
**Name**	**Start**	**End**	**Length (bp)**	**Start**	**End**	
Ca_18341	24514080	24515481	1,401	Ca1_8682204	Ca1_33504088	Heat shock protein
Ca_02777	10118666	10120375	1,709	Ca1_8682204	Ca1_33504088	Heat shock protein
Ca_06915	16486764	16488806	2,042	Ca1_8682204	Ca1_33504088	Alpha-crystallin domain of heat shock protein
Ca_24217	18375189	18377243	2,054	Ca1_8682204	Ca1_33504088	Pollen-specific leucine-rich repeat extensin-like protein 1
Ca_06900	16670592	16673062	2,470	Ca1_8682204	Ca1_33504088	Heat shock protein
Ca_02832	9661308	9664099	2,791	Ca1_8682204	Ca1_33504088	Heat shock 70 kDa protein
Ca_22117	21727236	21731181	3,945	Ca1_8682204	Ca1_33504088	Heat shock-like protein, putative
Ca_16007	17402067	17404092	2,025	Ca2_8484804	Ca2_31975221	Pollen-specific leucine-rich repeat extensin-like protein 1
Ca_24649	15993671	15997430	3,759	Ca2_8484804	Ca2_31975221	Truncated transcription factor CAULIFLOWER A-like
Ca_22033	16232678	16236688	4,010	Ca2_8484804	Ca2_31975221	Heat shock protein-binding protein
Ca_20135	22124562	22125716	1,154	Ca4_14861717	Ca4_40568556	Heat shock transcription factor A3
Ca_05385	17413769	17415327	1,558	Ca4_14861717	Ca4_40568556	Heat shock protein
Ca_25302	26380392	26382057	1,665	Ca4_14861717	Ca4_40568556	Heat shock protein
Ca_17137	20149055	20150949	1,894	Ca4_14861717	Ca4_40568556	Pollen-specific leucine-rich repeat extensin-like protein 1
Ca_22444	35651353	35653252	1,899	Ca4_14861717	Ca4_40568556	DnaJ heat shock amino-terminal domain protein
Ca_17160	20521283	20523227	1,944	Ca4_14861717	Ca4_40568556	Pollen-specific leucine-rich repeat extensin-like protein 1
Ca_20459	27199342	27201308	1,966	Ca4_14861717	Ca4_40568556	Heat shock protein
Ca_21304	27740485	27742509	2,024	Ca4_14861717	Ca4_40568556	Pollen-specific leucine-rich repeat extensin-like protein 1
Ca_15124	37590074	37592294	2,220	Ca4_14861717	Ca4_40568556	Pollen-specific leucine-rich repeat extensin-like protein 1
Ca_14182	30066661	30069132	2,471	Ca4_14861717	Ca4_40568556	DnaJ heat shock amino-terminal domain protein
Ca_14827	36505593	36508070	2,477	Ca4_14861717	Ca4_40568556	Pollen-specific LRR extensin-like protein
Ca_05401	17218852	17221940	3,088	Ca4_14861717	Ca4_40568556	DnaJ heat shock amino-terminal domain protein
Ca_14004	19020031	19024609	4,578	Ca4_14861717	Ca4_40568556	Pollen protein Ole E I-like protein
Ca_14192	30295389	30312868	17,479	Ca4_14861717	Ca4_40568556	Protein PHOTOPERIOD-INDEPENDENT EARLY FLOWERING 1 isoform X1
Ca_18924	28388535	28390538	2,003	Ca7_9555338	Ca7_47907019	Heat shock protein
Ca_16434	38877066	38879095	2,029	Ca7_9555338	Ca7_47907019	Pollen-specific leucine-rich repeat extensin-like protein 1
Ca_16239	26957077	26959156	2,079	Ca7_9555338	Ca7_47907019	Heat shock protein
Ca_16155	33278245	33280606	2,361	Ca7_9555338	Ca7_47907019	Pollen receptor-like kinase 3
Ca_10118	31400709	31404217	3,508	Ca7_9555338	Ca7_47907019	Protein EARLY FLOWERING 3
Ca_09277	12362555	12367223	4,668	Ca7_9555338	Ca7_47907019	Heat shock factor protein HSF24-like
Ca_13761	36747534	36754463	6,929	Ca7_9555338	Ca7_47907019	Calmodulin-binding heat-shock protein
Ca_17996	41387378	41400231	12,853	Ca7_9555338	Ca7_47907019	Flowering time control protein FY

## Discussion

In the context of climate change, every degree increase in aerial temperature has a severe impact on crop production, especially on chickpea that is predominantly cultivated on residual soil moisture in marginal environments (Gaur et al., 2014). Therefore, understanding the nature, impact, and molecular mechanisms of heat stress tolerance will help in designing strategies to overcome production losses. In chickpea, previously, very few studies were focused on understanding the nature, impact, and existing diversity in germplasm lines as well as identifying the genomic regions responsible to some extent. In this study, we reported major QTLs and novel genes in these genomic regions, which are associated with responsible for heat stress tolerance.

Late sowing, a simple and effective field screening technique for reproductive-stage heat tolerance in chickpea developed at the ICRISAT, Patancheru, India, was adopted for phenotyping the RILs for heat stress tolerance. The late sowing approach was adopted earlier in understanding the genetic variability for heat stress among genotypes as well as in identifying the genomic regions responsible for heat stress tolerance; for instance, in cool season crops, namely, chickpea (Paul et al., 2018b), wheat (Sareen et al., 2020), brassica (Branham et al., 2017), and rice (Prasanth et al., 2016). As the selection was based on yield *per se* results in a slower response because of genotype × environmental interactions, we also phenotyped the mapping population for physiological traits like CMS, NDVI, NBI, and CHL, which could be used as an indirect selection criterion to improve heat tolerance in chickpea as this was used in other crop plants. A sufficient amount of genetic variability for various phenological, physiological, and yield-related traits was noted in both the parents, and the RIL population was studied under NS and HTS environments for both years. The similar results were also reported earlier in chickpea based on evaluating the germplasm lines as well as one RIL population under HTS environment (Krishnamurthy et al., 2011; Devasirvatham et al., 2013; Paul et al., 2018b). High heritability for physiological traits like CMS, NDVI, NBI, and CHL contents under HTS environments indicates that the selection for heat tolerance relying on these traits could be effective. Earlier, heat stress was reported to reduce the total CHL and showed moderate to high heritability for NDVI, CMS, and CHL content under stress condition in wheat (Bhusal et al., 2018; Condorelli et al., 2018; Pradhan et al., 2020), maize (Naveed et al., 2016), carrot (Nijabat et al., 2020), and pea (Tafesse et al., 2020). Low heritability for HI trait under HTS environments observed in this study indicates that the election for this trait will not enhance yield under stress. As yield traits remain the primary objective for improving the heat tolerance in all crop plants including chickpea, a positive and significant correlation among the yield and yield-related traits especially, FP, SYPP, and BYPP could serve as an important parameter for developing heat-tolerant chickpea genotype.

In this study, using a RIL population with a dense genetic map and phenotyping under NS and HTS allowed us to precisely identify major QTLs for heat stress in chickpea. Our genetic map has approximately 3-fold more markers compared to the previous study reporting the QTLs for heat stress tolerance (Paul et al., 2018a). In addition to reporting QTLs for phenological, yield, and yield-related traits under heat stress environments, to the best of our knowledge, this study is the first comprehensive study that reports QTLs for physiological traits like CHL, NBI, NDVI, CMS, and NPP in chickpea. A better understanding of phenology in response to heat stress will enable designing the breeding strategies. Minor QTLs for DFI were identified on CaLG06 and CaLG08, while a major QTL was identified for DM on CaLG01. Physiological traits like CMS, NDVI, and NBI, to date, have been used as proxy for grain yield under stress mostly in cereals (ElBasyoni et al., 2017; Bhusal et al., 2018; Condorelli et al., 2018; Getahun et al., 2020; Khanna-Chopra and Semwal, 2020). In this study, we reported major QTLs for these traits in chickpea, which may be used, after validation, for marker-assisted breeding for heat stress tolerance in chickpea.

In the case of chickpea, four flowering time genes (*efl-1* from ICCV 96029, *efl-3* from BGD 132, and *efl-4* from ICC 16641) and their allelic relationships were reported (Gaur et al., 2015), and major QTLs corresponding to these genes were mapped on CaLG04, CaLG08, and CaLG06, respectively (Mallikarjuna et al., 2017). Furthermore, marker trait associations for flowering time were reported earlier (Thudi et al., 2014; Upadhyaya et al., 2015; Varshney et al., 2019). However, in this study, we reported QTLs for DFI on CaLG06 and CaLG08 under heat stress environments as well as based on the pooled data of 2 years. Furthermore, interestingly, QTLs for DPI and DFI co-localized or mapped in the same genomic region under HTS environments of both years as well as based on pooled data. These observations indicate that introgression of one of the traits simultaneously improves both the traits, which are key for achieving resilience to heat stress.

Earlier two major genomic regions harboring QTLs for heat tolerance-related traits were mapped on CaLG05 and CaLG06; however, none on the QTLs explained >20% PVE (Paul et al., 2018a). Nevertheless, HI in this study explained >30% PVE. Similarly, CaLG04 also harbored QTLs for five traits (CHL, CMS, NDVI, DPF, and 100SDW), among these QTLs for traits CHL and 100SDW explained >30% PVE. Except HI (PVE 39.13%, NS 2018), none of the QTLs mapped on CaLG06 had PVE >15%. Similarly, QTLs for traits like CHL, NDVI, and HI were mapped on CaLG05 which explained 6.78–18.69% PVE. Earlier, a genomic region refereed as “*QTL-hotspot*” was reported to harbor several QTLs for different drought tolerance-related traits including 100SDW on CaLG04 (Varshney et al., 2014). A genomic region on CaLG07 harbors QTLs explaining >30% PVE for DPI and 100SDW as well as QTL for NBI explaining >10% PVE. For 100SWD, a total of four major QTLs were identified on CaLG01, CaLG04, and CaLG07 under HTS, and no QTLs were detected under NS. For SYPP trait, two major QTLs were identified on CaLG02 and CaLG06 under HTS. Only one QTL was identified on CaLG06 under NS. However, yield-related QTLs were not consistently recorded under all the conditions suggesting their environmental specific expression. Likewise, QTLs contributing to pods per plant, seed yield per plant, and seed weight were reported on CaLG01 and CaLG06 (Bajaj et al., 2015; Srivastava et al., 2016). In the case of cowpea, four QTLs were identified for pod set number per peduncle under HS and markers, which were utilized in breeding applications (Lucas et al., 2013; Pottorff et al., 2014). Similarly, in lentils, a major QTL controlling the seedling survival and pod setting traits under heat stress was noticed (Singh et al., 2017). In addition, QTLs for SYPP and BYPP (full names) were mapped in the same genomic region under NS environments of both years as well as based on pooled data. For yield and yield-related traits like DPI, DPF, and SYPP under HTS major alleles were contributed by ICCV 92944. For the 100SDW trait under HTS major alleles were contributed by DCP 92-3. On the other hand, almost all of the traits DPI, BYPP, and SYPP were contributed by DCP 92-3 under NS. For the trait HI under NS major alleles was contributed by both parents. In addition to these major QTLs, several QTLs were identified that were environmentally specific under NS and HTS, which only appeared in this study in the first year (NS or HTS). A total of nine major QTLs were located on CaLG06, which highlight the importance of this region in the heat tolerance mechanism in chickpea. Some QTLs were largely affected by environmental factors and that could be detected in only one season, and for these QTLs, further verification should be required.

*HSP* genes play a pivotal role in heat stress tolerance. In this study, 32 genes were identified in the QTL regions of CaLG01, CaLG02, CaLG04, and CaLG07. Similarly, in the case of soybean, 38 Hsfs were identified that were located on 15 soybean chromosomes (Li et al., 2014). *HSP* gene families were reported to be involved in drought and heat stress responses in soybean seedlings (Zhang et al., 2015; Das et al., 2016; Liu et al., 2019). *HSP90* gene families in five legumes and expression profiles in chickpea were reported earlier (Agarwal et al., 2016). Furthermore, based on genome-wide association studies, especially eight flowering time-regulating genes (*efl1, FLD, GI, Myb, SFH3, bZIP, bHLH*, and *SBP*) were reported (Upadhyaya et al., 2015). The genes reported in this study can be further explored for haplotypes based on the germplasm sequence information available in the public domain that has the potential for genetic improvement of the trait (Varshney et al., 2020). In addition, pollen-specific leucine-rich repeat extensin-like protein 1 genes identified in the QTL regions were reported to synergistically maintain pollen tube cell wall integrity; thus, they play critical roles in pollen germination and pollen tube growth (Wang et al., 2018). Recently, cloning of SHY in tomato, a pollen-specific gene that encodes a leucine-rich repeat (LRR) protein, demonstrated its role in a signal transduction pathway mediating pollen tube growth (Guyon et al., 2004).

## Conclusions

In this study, we identified a total of 37 major QTLs across the genome for 12 traits. DFI, DPI, and DM are the key traits for escaping the heat stress in chickpea especially reproductive heat stress that hampers chickpea production. In this study, we reported major QTLs explaining >30% PVE for these key traits that contribute to yield under heat stress. In addition, we also reported for the first time major QTLs for proxy traits (physiological traits like CHL, NBI, NDVI, and CMS). Furthermore, 32 candidate genes in the QTL regions that encode the *HSP*, heat shock transcription factors, genes are involved in flowering time regulation as well as pollen-specific genes. The major QTLs reported in this study may be useful in molecular breeding for developing heat-tolerant superior lines or varieties.

## Data Availability Statement

The original contributions presented in the study are publicly available. This data can be found here: NCBI repository, BioProject ID PRJNA695065 (http://www.ncbi.nlm.nih.gov/bioproject/695065).

## Author Contributions

UJ and MT conceived the idea. UJ, HN, and RJ conducted the experiments. UJ performed the statistical analysis. MT, UJ, RP, and RV prepared the manuscript. VV and PB performed the GBS analysis. NS contributed to the development and phenotyping of mapping population. AC and RV contributed to consumables and the generation of genotyping data. All authors read and approved the final manuscript.

## Conflict of Interest

The authors declare that the research was conducted in the absence of any commercial or financial relationships that could be construed as a potential conflict of interest.

## Publisher's Note

All claims expressed in this article are solely those of the authors and do not necessarily represent those of their affiliated organizations, or those of the publisher, the editors and the reviewers. Any product that may be evaluated in this article, or claim that may be made by its manufacturer, is not guaranteed or endorsed by the publisher.

## Supplementary Material

Supplementary Figure 1 Mean weekly minimum and maximum day temperature (°C) recorded during the crop season of 2017–2018 and 2018–2019. Temperature >30°C during reproductive stages especially at podding stage and pod filling were recorded, which is more than the critical temperatures that hamper the production of chickpea.

Supplementary Figure 2 High density genetic map comprising 788 single-nucleotide polymorphism markers mapped on eight chromosomes of chickpea. The map distance is indicated as a common scale for all eight linkage groups (CaLG01–CaLG08) on the left side figure and marker names on the right side of each linkage group.

Supplementary Table 1 Correlations among different traits under normal sown environments of 2017–2018.

Supplementary Table 2 Correlations among different traits under late sown or heat stress environments of 2017–2018.

Supplementary Table 3 Correlations among different traits under normal sown environments of 2018–2019.

Supplementary Table 4 Correlations among different traits under late sown or heat stress environments of 2018–2019.

Supplementary Table 5 Correlations between different traits based on pooled data of 2 years under normal sown environments.

Supplementary Table 6 Correlations between different traits based on pooled data of 2 years under late sown or heat stress environments.

Supplementary Table 7 Genotyping-by-sequencing–single-nucleotide polymorphism (GBS–SNP) marker data used for construction of genetic map for recombinant inbred line population derived from DCP 92-3 (heat sensitive) × ICCV 92944 (heat tolerant).

Supplementary Table 8 Distribution of SNPs on the genetic map derived from DCP 92-3 × ICCV 92944.

Supplementary Table 9 Genes identified in the genomic regions harboring major QTLs on CaLG01, CaLG02, CaLG04, CaLG06, and CaLG07.
