# Supplementary figures and images for: Major QTLs and Potential Candidate Genes for Heat Stress Tolerance Identified in Chickpea (Cicer arietinum L.)
Source: Front Plant Sci. 2021 Jul 26;12:655103. doi: 10.3389/fpls.2021.655103 (PMC8350164; doi:10.3389/fpls.2021.655103)

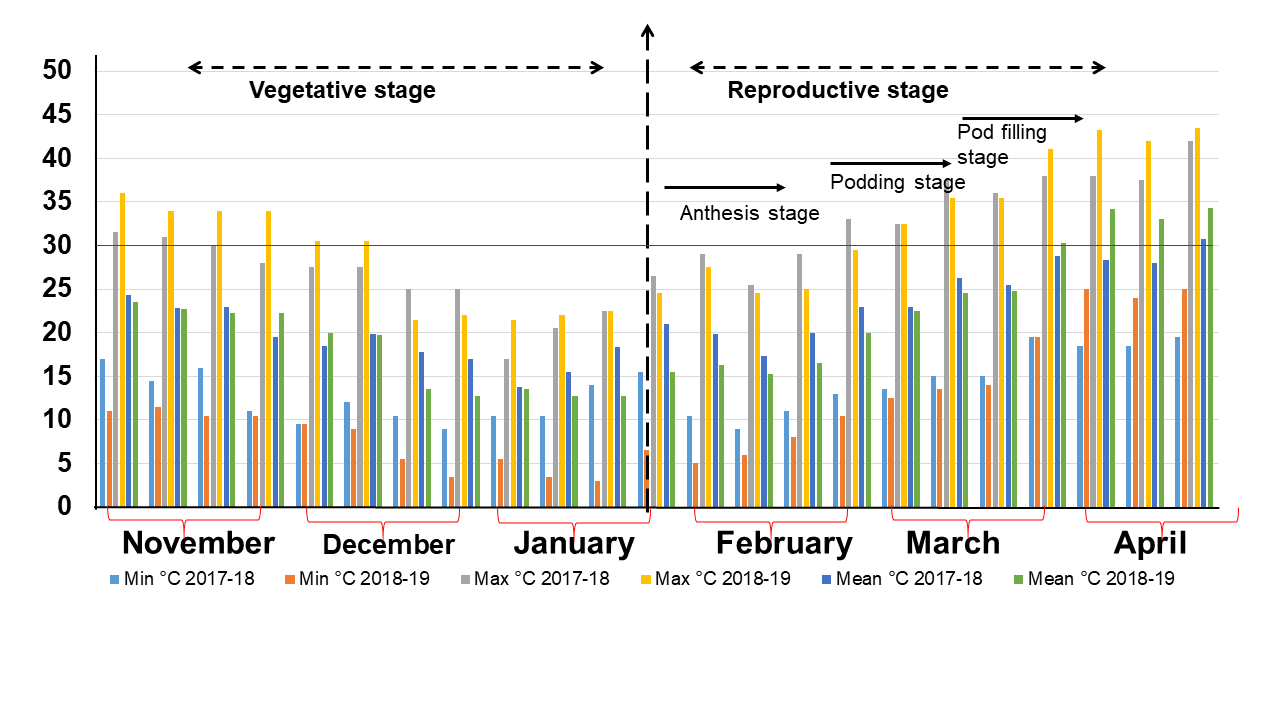

Supplement: Supplementary Figure 1 — Mean weekly minimum and maximum day temperature (°C) recorded during the crop season of 2017–2018 and 2018–2019. Temperature >30°C during reproductive stages especially at podding stage and pod filling were recorded, which is more than the critical temperatures that hamper the production of chickpea. [file Image_1.tif]

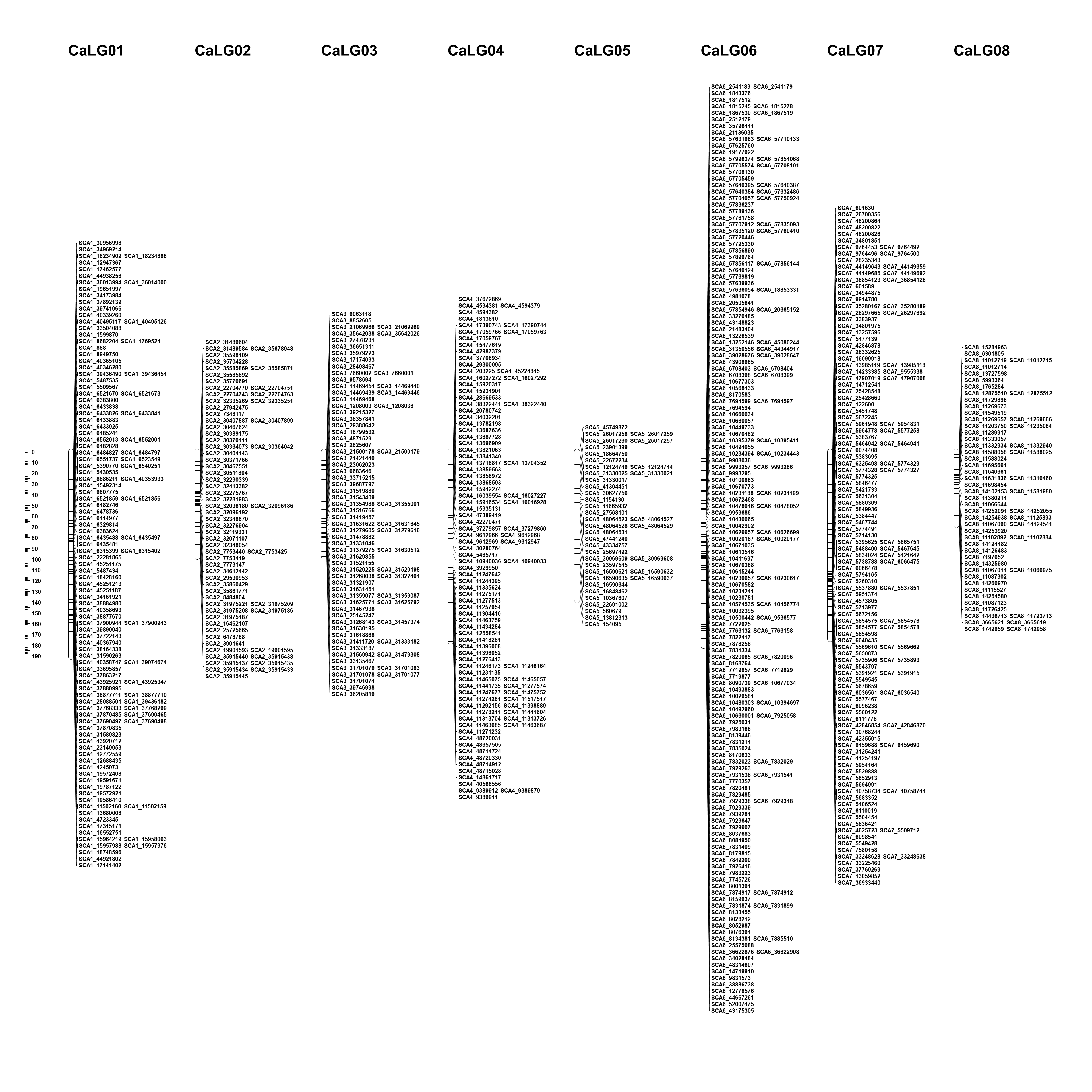

Supplement: Supplementary Figure 2 — High density genetic map comprising 788 single-nucleotide polymorphism markers mapped on eight chromosomes of chickpea. The map distance is indicated as a common scale for all eight linkage groups (CaLG01–CaLG08) on the left side figure and marker names on the right side of each linkage group. [file Image_2.tif]
