# Supplementary material for: Major QTLs and Potential Candidate Genes for Heat Stress Tolerance Identified in Chickpea (Cicer arietinum L.)
Source: Front Plant Sci. 2021 Jul 26;12:655103. doi: 10.3389/fpls.2021.655103 (PMC8350164; doi:10.3389/fpls.2021.655103)
Supplement: Supplementary Table 1 — Correlations among different traits under normal sown environments of 2017–2018. [file Table_1.DOCX]

**Supplementary Table 1: Correlations among different traits under normal sown environments of 2017-18**

| **Traits** | **DFI (d)** | **DPI (d)** | **DPF (d)** | **DM (d)** | **NDVI** | **NBI** | **CHL (ng/ mm^2^)** | **CMS (%)** | **FP** | **BYPP (g)** | **100SDW (g)** | **HI (%)** | **SYPP (g)** |
| --- | --- | --- | --- | --- | --- | --- | --- | --- | --- | --- | --- | --- | --- |
| **DFI (d)** | 1.00 |  |  |  |  |  |  |  |  |  |  |  |  |
| **DPI (d)** | 0.72^**^ | 1.00 |  |  |  |  |  |  |  |  |  |  |  |
| **DPF (d)** | -0.24 | -0.40 | 1.00 |  |  |  |  |  |  |  |  |  |  |
| **DM(d)** | 0.58^**^ | 0.39^**^ | 0.09 | 1.00 |  |  |  |  |  |  |  |  |  |
| **NDVI** | -0.05 | -0.06 | 0.05 | -0.09 | 1.00 |  |  |  |  |  |  |  |  |
| **NBI** | -0.18 | -0.22 | 0.08 | -0.01 | -0.03 | 1.00 |  |  |  |  |  |  |  |
| **CHL (ng/ mm^2^)** | -0.08 | -0.11 | 0.06 | -0.16 | -0.04 | 0.01 | 1.00 |  |  |  |  |  |  |
| **CMS (%)** | 0.02 | 0.02 | -0.05 | -0.03 | -0.09 | -0.10 | 0.01 | 1.00 |  |  |  |  |  |
| **FP** | 0.13 | 0.15 | -0.09 | -0.01 | -0.11 | -0.13 | 0.13 | 0.15 | 1.00 |  |  |  |  |
| **BYPP (g)** | -0.17 | -0.20 | -0.08 | -0.07 | -0.12 | -0.03 | 0.00 | -0.08 | 0.30^*^ | 1.00 |  |  |  |
| **100SDW (g)** | -0.05 | 0.01 | -0.11 | -0.09 | 0.05 | -0.02 | -0.09 | -0.04 | -0.09 | 0.36^*^ | 1.00 |  |  |
| **HI (%)** | 0.04 | 0.00 | -0.02 | 0.13 | -0.09 | 0.02 | 0.01 | 0.05 | 0.14 | 0.33^*^ | 0.32^*^ | 1.00 |  |
| **SYPP (g)** | -0.06 | -0.07 | -0.10 | -0.06 | -0.07 | -0.06 | 0.05 | 0.02 | 0.57^**^ | 0.61^**^ | 0.63^**^ | 0.38^*^ | 1.00 |

DFI- days to flower initiation; DPI- days to pod initiation; DPF- days to pod filling; DM- days to maturity; NDVI-normalized difference vegetation index; NBI-nitrogen balance index; CHL-chlorophyll content; CMS-cell membrane stability; FP-filled pods; BYPP-biological plant yield, 100SDW- 100 seed weight; HI- harvest index, SYPP seed yield per plant.

^*^Significant at 0.05%

^**^ Significant at 0.01%

**Supplementary Table 2: Correlations among different traits under late sown or heat stress environments of 2017-18**

| **Traits** | **DFI (d)** | **DPI (d)** | **DPF (d)** | **DM (d)** | **NDVI** | **NBI** | **CHL (ng/ mm^2^)** | **CMS (%)** | **FP** | **BYPP (g)** | **100SDW (g)** | **HI (g)** | **SYPP (g)** |
| --- | --- | --- | --- | --- | --- | --- | --- | --- | --- | --- | --- | --- | --- |
| **DFI (d)** | 1.00 |  |  |  |  |  |  |  |  |  |  |  |  |
| **DPI (d)** | 0.82^**^ | 1.00 |  |  |  |  |  |  |  |  |  |  |  |
| **DPF (d)** | -0.02 | -0.19 | 1.00 |  |  |  |  |  |  |  |  |  |  |
| **DM(d)** | 0.69^**^ | 0.68^**^ | 0.02 | 1.00 |  |  |  |  |  |  |  |  |  |
| **NDVI** | -0.05 | -0.03 | -0.02 | 0.06 | 1.00 |  |  |  |  |  |  |  |  |
| **NBI** | -0.07 | -0.14 | 0.05 | -0.01 | 0.09 | 1.00 |  |  |  |  |  |  |  |
| **CHL (ng/ mm^2^)** | -0.02 | -0.06 | 0.00 | -0.06 | 0.02 | 0.52^**^ | 1.00 |  |  |  |  |  |  |
| **CMS (%)** | 0.00 | -0.01 | 0.01 | -0.09 | -0.15 | -0.04 | 0.07 | 1.00 |  |  |  |  |  |
| **FP** | -0.06 | -0.03 | 0.01 | -0.06 | 0.13 | 0.01 | -0.01 | 0.19 | 1.00 |  |  |  |  |
| **BYPP (g)** | 0.02 | 0.03 | -0.02 | 0.01 | -0.02 | -0.08 | -0.01 | 0.15 | 0.42^**^ | 1.00 |  |  |  |
| **100SDW (g)** | -0.09 | -0.01 | -0.05 | -0.05 | 0.01 | 0.00 | 0.01 | 0.09 | 0.03 | 0.34^*^ | 1.00 |  |  |
| **HI (%)** | -0.15 | -0.14 | -0.01 | -0.13 | 0.10 | 0.12 | -0.03 | 0.06 | 0.25 | -0.27 | 0.38^*^ | 1.00 |  |
| **SYPP (g)** | -0.09 | -0.06 | -0.02 | -0.09 | 0.05 | 0.03 | -0.01 | 0.16 | 0.57^**^ | 0.59^**^ | 0.64^**^ | 0.57^**^ | 1.00 |

DFI- days to flower initiation; DPI- days to pod initiation; DPF- days to pod filling; DM- days to maturity; NDVI-normalized difference vegetation index; NBI-nitrogen balance index; CHL-chlorophyll content; CMS-cell membrane stability; FP-filled pods; BYPP-biological plant yield, 100SDW- 100 seed weight; HI- harvest index, SYPP seed yield per plant.

^*^Significant at 0.05%

^**^ Significant at 0.01%

**Supplementary Table 3: Correlations among different traits under normal sown environments of 2018-19**

| **Traits** | **DFI (d)** | **DPI (d)** | **DPF (d)** | **DM (d)** | **NDVI** | **NBI** | **CHL (ng/ mm^2^)** | **CMS (%)** | **FP** | **BYPP (g)** | **100SDW (g)** | **HI (g)** | **SYPP (g)** |
| --- | --- | --- | --- | --- | --- | --- | --- | --- | --- | --- | --- | --- | --- |
| **DFI (d)** | 1.00 |  |  |  |  |  |  |  |  |  |  |  |  |
| **DPI (d)** | 0.65^**^ | 1.00 |  |  |  |  |  |  |  |  |  |  |  |
| **DPF (d)** | -0.17 | -0.36 | 1.00 |  |  |  |  |  |  |  |  |  |  |
| **DM(d)** | 0.40^*^ | 0.51^**^ | -0.04 | 1.00 |  |  |  |  |  |  |  |  |  |
| **NDVI** | 0.14 | 0.09 | -0.03 | 0.18 | 1.00 |  |  |  |  |  |  |  |  |
| **NBI** | 0.00 | 0.00 | -0.01 | 0.01 | 0.19 | 1.00 |  |  |  |  |  |  |  |
| **CHL (ng/ mm^2^)** | -0.04 | -0.07 | 0.04 | -0.12 | -0.03 | -0.04 | 1.00 |  |  |  |  |  |  |
| **CMS (%)** | -0.06 | -0.09 | -0.01 | -0.02 | 0.05 | -0.01 | -0.04 | 1.00 |  |  |  |  |  |
| **FP** | 0.41^**^ | 0.36 | -0.11 | 0.11 | 0.03 | -0.05 | 0.06 | 0.02 | 1.00 |  |  |  |  |
| **BYPP (g)** | 0.18 | 0.19 | -0.13 | 0.03 | 0.06 | -0.01 | 0.01 | 0.01 | 0.52^**^ | 1.00 |  |  |  |
| **100SDW (g)** | -0.03 | 0.05 | -0.08 | 0.03 | 0.01 | 0.04 | -0.09 | -0.08 | 0.03 | 0.72^**^ | 1.00 |  |  |
| **HI (%)** | 0.12 | 0.10 | -0.07 | 0.15 | 0.09 | 0.05 | -0.04 | -0.01 | 0.41^**^ | 0.21 | 0.40^**^ | 1.00 |  |
| **SYPP (g)** | 0.21 | 0.21 | -0.13 | 0.09 | 0.09 | 0.01 | -0.02 | 0.00 | 0.60^**^ | 0.91^**^ | 0.76^**^ | 0.58^**^ | 1.00 |

DFI- days to flower initiation; DPI- days to pod initiation; DPF- days to pod filling; DM- days to maturity; NDVI-normalized difference vegetation index; NBI-nitrogen balance index; CHL-chlorophyll content; CMS-cell membrane stability; FP-filled pods; BYPP-biological plant yield, 100SDW- 100 seed weight; HI- harvest index, SYPP seed yield per plant.

^*^Significant at 0.05%

^**^ Significant at 0.01%

**Supplementary Table 4: Correlations among different traits under late sown or heat stress environments of 2017-18**

| **Traits** | **DFI (d)** | **DPI (d)** | **DPF (d)** | **DM (d)** | **NDVI** | **NBI** | **CHL(ng/ mm^2^)** | **CMS (%)** | **FP** | **BYPP (g)** | **100SDW (g)** | **HI (g)** | **SYPP (g)** |
| --- | --- | --- | --- | --- | --- | --- | --- | --- | --- | --- | --- | --- | --- |
| **DFI (d)** | 1.00 |  |  |  |  |  |  |  |  |  |  |  |  |
| **DPI (d)** | 0.87^**^ | 1.00 |  |  |  |  |  |  |  |  |  |  |  |
| **DPF (d)** | -0.09 | -0.06 | 1.00 |  |  |  |  |  |  |  |  |  |  |
| **DM(d)** | 0.33^*^ | 0.38^*^ | -0.03 | 1.00 |  |  |  |  |  |  |  |  |  |
| **NDVI** | -0.10 | -0.10 | 0.00 | -0.12 | 1.00 |  |  |  |  |  |  |  |  |
| **NBI** | 0.17 | 0.19 | -0.02 | 0.20 | -0.08 | 1.00 |  |  |  |  |  |  |  |
| **CHL (ng/ mm^2^)** | 0.12 | 0.10 | -0.09 | 0.11 | -0.01 | -0.02 | 1.00 |  |  |  |  |  |  |
| **CMS (%)** | 0.08 | 0.09 | -0.06 | 0.06 | -0.17 | 0.08 | 0.18 | 1.00 |  |  |  |  |  |
| **FP** | 0.08 | 0.07 | -0.06 | 0.07 | 0.00 | 0.11 | 0.16 | 0.21 | 1.00 |  |  |  |  |
| **BYPP (g)** | 0.10 | 0.14 | -0.02 | 0.04 | -0.14 | 0.06 | 0.17 | 0.18 | 0.26^*^ | 1.00 |  |  |  |
| **100SDW (g)** | -0.06 | 0.01 | -0.05 | -0.05 | -0.01 | -0.03 | -0.03 | 0.06 | 0.07 | 0.15 | 1.00 |  |  |
| **HI (%)** | -0.05 | -0.05 | -0.03 | 0.01 | 0.07 | -0.02 | 0.00 | 0.08 | 0.52^**^ | -0.32 | 0.55^**^ | 1.00 |  |
| **SYPP (g)** | 0.11 | 0.13 | -0.12 | 0.10 | -0.07 | 0.12 | 0.17 | 0.27^*^ | 0.75^**^ | 0.32^*^ | 0.63^**^ | 0.68^**^ | 1.00 |

DFI- days to flower initiation; DPI- days to pod initiation; DPF- days to pod filling; DM- days to maturity; NDVI-normalized difference vegetation index; NBI-nitrogen balance index; CHL-chlorophyll content; CMS-cell membrane stability; FP-filled pods; BYPP-biological plant yield, 100SDW- 100 seed weight; HI- harvest index, SYPP seed yield per plant.

^*^Significant at 0.05%

^**^ Significant at 0.01%

**Supplementary Table 5: Correlations between different traits based on pooled data of two years under normal sown environments**

| **Traits** | **DFI (d)** | **DPI (d)** | **DPF (d)** | **DM (d)** | **NDVI** | **NBI** | **CHL (ng/ mm^2^)** | **CMS (%)** | **FP** | **BYPP (g)** | **100SDW (g)** | **HI (g)** | **SYPP (g)** |
| --- | --- | --- | --- | --- | --- | --- | --- | --- | --- | --- | --- | --- | --- |
| **DFI (d)** | 1.00 |  |  |  |  |  |  |  |  |  |  |  |  |
| **DPI (d)** | 0.65** | 1.00 |  |  |  |  |  |  |  |  |  |  |  |
| **DPF (d)** | -0.25 | -0.36 | 1.00 |  |  |  |  |  |  |  |  |  |  |
| **DM(d)** | 0.54** | 0.46** | 0.01 | 1.00 |  |  |  |  |  |  |  |  |  |
| **NDVI** | -0.10 | -0.08 | 0.03 | -0.14 | 1.00 |  |  |  |  |  |  |  |  |
| **NBI** | -0.07 | -0.08 | 0.00 | -0.04 | -0.15 | 1.00 |  |  |  |  |  |  |  |
| **CHL(ng/ mm^2^)** | -0.02 | -0.15 | 0.02 | -0.14 | 0.04 | -0.04 | 1.00 |  |  |  |  |  |  |
| **CMS (%)** | -0.02 | -0.04 | 0.00 | 0.00 | -0.11 | -0.01 | -0.04 | 1.00 |  |  |  |  |  |
| **NPP** | 0.34* | 0.19 | -0.15 | 0.08 | -0.13 | -0.12 | 0.14 | 0.15 | 1.00 |  |  |  |  |
| **BYPP (g)** | 0.12 | 0.05 | -0.20 | 0.01 | -0.11 | -0.06 | 0.05 | -0.02 | 0.43^**^ | 1.00 |  |  |  |
| **100SDW (g)** | -0.05 | 0.05 | -0.11 | -0.03 | -0.04 | 0.00 | -0.12 | -0.06 | -0.06 | 0.65^**^ | 1.00 |  |  |
| **HI (%)** | 0.11 | 0.06 | -0.06 | 0.19 | -0.13 | 0.03 | -0.02 | 0.07 | 0.33^*^ | 0.36^*^ | 0.44^**^ | 1.00 |  |
| **SYPP (g)** | 0.16 | 0.05 | -0.17 | 0.06 | -0.13 | -0.07 | 0.03 | 0.06 | 0.55^**^ | 0.82^**^ | 0.74^**^ | 0.60^**^ | 1.00 |

DFI- days to flower initiation; DPI- days to pod initiation; DPF- days to pod filling; DM- days to maturity; NDVI-normalized difference vegetation index; NBI-nitrogen balance index; CHL-chlorophyll content; CMS-cell membrane stability; FP-filled pods; BYPP-biological plant yield, 100SDW- 100 seed weight; HI- harvest index, SYPP seed yield per plant.

**Supplementary Table 6: Correlations between different traits based on pooled data of two years under heat stress environments**

| **Traits** | **DFI (d)** | **DPI (d)** | **DPF (d)** | **DM (d)** | **NDVI** | **NBI** | **CHL (ng/ mm^2^)** | **CMS (%)** | **FP** | **BYPP (g)** | **100SDW (g)** | **HI (g)** | **SYPP (g)** |
| --- | --- | --- | --- | --- | --- | --- | --- | --- | --- | --- | --- | --- | --- |
| **DFI (d)** | 1.00 |  |  |  |  |  |  |  |  |  |  |  |  |
| **DPI (d)** | 0.84^**^ | 1.00 |  |  |  |  |  |  |  |  |  |  |  |
| **DPF (d)** | -0.05 | -0.12 | 1.00 |  |  |  |  |  |  |  |  |  |  |
| **DM(d)** | 0.57^**^ | 0.61^**^ | 0.03 | 1.00 |  |  |  |  |  |  |  |  |  |
| **NDVI** | -0.09 | -0.07 | -0.02 | -0.10 | 1.00 |  |  |  |  |  |  |  |  |
| **NBI** | -0.07 | -0.11 | 0.08 | -0.10 | -0.04 | 1.00 |  |  |  |  |  |  |  |
| **CHL (ng/ mm^2^)** | -0.03 | -0.05 | -0.05 | -0.09 | 0.11 | 0.46^**^ | 1.00 |  |  |  |  |  |  |
| **CMS (%)** | 0.00 | 0.01 | -0.02 | -0.07 | -0.05 | 0.00 | 0.05 | 1.00 |  |  |  |  |  |
| **NPP** | -0.01 | -0.01 | -0.02 | -0.02 | 0.11 | -0.04 | 0.00 | 0.13 | 1.00 |  |  |  |  |
| **BYPP (g)** | 0.08 | 0.11 | 0.01 | 0.06 | -0.13 | -0.02 | 0.01 | 0.07 | 0.32^*^ | 1.00 |  |  |  |
| **100SDW (g)** | -0.07 | 0.02 | -0.03 | -0.01 | 0.02 | -0.01 | -0.01 | 0.04 | 0.06 | 0.28 | 1.00 |  |  |
| **HI (%)** | -0.10 | -0.09 | -0.03 | -0.08 | 0.13 | 0.01 | -0.04 | 0.04 | 0.43^**^ | -0.28 | 0.54^**^ | 1.00 |  |
| **SYPP (g)** | 0.00 | 0.04 | -0.06 | -0.02 | 0.03 | -0.02 | -0.03 | 0.10 | 0.65^**^ | 0.46 | 0.72^**^ | 0.68^**^ | 1.00 |

DFI- days to flower initiation; DPI- days to pod initiation; DPF- days to pod filling; DM- days to maturity; NDVI-normalized difference vegetation index; NBI-nitrogen balance index; CHL-chlorophyll content; CMS-cell membrane stability; FP-filled pods; BYPP-biological plant yield, 100SDW- 100 seed weight; HI- harvest index, SYPP seed yield per plant

^*^Significant at 0.05%

^**^ Significant at 0.01%

**Supplementary Table 8: Distribution of SNPs on the genetic map derived from DCP 92-3 × ICCV 92944**

| **Linkage group** | **Number of**  **SNPs**  **identified** | **Filtered SNPs**  **used for**  **mapping** | **Number of**  **SNPs mapped** | **Genetic distance**  **(cM)** | **Inter marker distance**  **(cM)** |
| --- | --- | --- | --- | --- | --- |
| CaLG01 | 1170 | 577 | 122 | 191 | 1.57 |
| CaLG02 | 848 | 412 | 67 | 98 | 1.46 |
| CaLG03 | 816 | 466 | 79 | 98 | 1.24 |
| CaLG04 | 1063 | 550 | 100 | 177 | 1.77 |
| CaLG05 | 955 | 526 | 40 | 138 | 3.45 |
| CaLG06 | 1465 | 691 | 185 | 181 | 0.98 |
| CaLG07 | 1314 | 639 | 135 | 174 | 1.29 |
| CaLG08 | 316 | 173 | 60 | 68 | 1.13 |
| **Total** | **7947** | **4034** | **788** | **1125** | **1.61** |
